# Impacts of different treatment methods on patients with COVID-related olfactory dysfunction: a systematic review and network meta-analysis

**DOI:** 10.1016/j.clinsp.2026.101103

**Published:** 2026-09-08

**Authors:** Wenli You, Lixin Luo, Ping Zhou, Tingting He, Mincong Shao, Tuoquan Jiao, Xuemei An

**Affiliations:** a School of Nursing, Chengdu University of Traditional Chinese Medicine, Chengdu City, Sichuan Province, China; b Hospital of Chengdu University of Traditional Chinese Medicine, Chengdu City, Sichuan Province, China; c Deyang Hospital Affiliated to Chengdu University of Traditional Chinese Medicine, Deyang City, Sichuan Province, China

**Keywords:** COVID-19, Olfactory dysfunction, Olfactory training, Network meta-analysis

## Abstract

**Background:**

Olfactory Dysfunction (OD) is a prevalent neurological sequela of Coronavirus Disease 2019 (COVID-19) and substantially impairs quality of life. Although multiple pharmaceutical and non-pharmaceutical interventions have been proposed, their relative efficacy remains controversial. This study aimed to compare the efficacy of existing interventions for post-COVID-19 OD.

**Methods:**

PubMed, Embase, the Cochrane Library, and Web of Science databases were systematically searched up to July 31, 2025. Randomized Controlled Trials (RCTs), cohort studies, and non-RCT studies evaluating interventions for post-COVID-19 OD were included. Study quality was assessed using the National Institutes of Health (NIH) scale and the Newcastle-Ottawa Scale (NOS). Data were analyzed using RStudio and Stata. The interventions were ranked by Surface Under the Cumulative Ranking (SUCRA) values.

**Results:**

A total of 44 studies involving 3,516 patients and 26 interventions were included, with overall moderate to high quality. Chelators (Standardized Mean Difference [SMD = 3.61], 95% Credible Interval [CrI]: 2.77, 4.55) significantly improved total olfactory scores and their subdomains (with SUCRA values of 98.2%). Platelet-rich plasma (SMD = 1.33, 95% CrI: 0.92, 1.74) markedly improved the results of the University of Pennsylvania Smell Identification Test (UPSIT). Corticosteroids substantially improved olfactory function, as revealed by the Visual Analogue Scale (VAS) (SMD = 1.99, 95% CrI: 0.52, 3.48). Functional Septorhinoplasty (FSPR) demonstrated superior improvement in sinonasal-related quality of life, as measured by the 22-item Sino-nasal Outcome Test (SNOT-22) (SMD = -1.06, 95% CrI: -1.91, -0.21).

**Conclusion:**

Chelators and chelators combined with other interventions demonstrated benefit in improving post-COVID-19 olfactory function, offering new directions for multi-target therapies. Further high-quality RCTs are required to confirm these findings.

## Highlights

• Chelating have shown good efficacy in improving overall olfactory function.
• PRP and OT + VitA improved odor identification ability.
• Corticosteroids and FSPR improved subjective symptoms and quality of life.

## Introduction

Coronavirus Disease 2019 (COVID-19) has spread rapidly since its outbreak in late 2019, posing a substantial threat to global public health.^1^ Olfactory Dysfunction (OD) is recognized as one of the most common neurological manifestations of COVID-19, primarily presenting as hyposmia, anosmia, and parosmia.^1^ Studies have indicated that approximately 5%–85% of COVID-19 patients experience OD during infection, with symptoms persisting for months in some cases.^2^, ^3^, ^4^ Prolonged OD not only leads to hypogeusia, diminished interest in eating and drinking, and changes in body weight, but also greatly elevates the risk of anxiety, depression, and social isolation, severely compromising patients' quality of life.^5^^,^^6^

The pathogenesis of OD is complex. Current research suggests it primarily results from the direct damage to olfactory epithelial supporting cells and olfactory bulb neurons caused by Severe Acute Respiratory Syndrome Coronavirus-2 (SARS-CoV-2), as well as local inflammatory responses, vascular injury, and impaired neural plasticity.^7^ As OD serves as an early clinical marker of COVID-19 infection, it is clinically important to detect OD early and give timely interventions. However, no consensus has been reached on the diagnosis and treatment of COVID-19-related OD. Treatment strategies are largely drawn from experience in post-viral OD treatment, including pharmaceutical therapies such as systemic or topical glucocorticoids, Alpha-Lipoic Acid (ALA), Vitamin A (VitA), and non-pharmaceutical therapies such as Olfactory Training (OT).^8^, ^9^, ^10^, ^11^ Among them, OT is widely recommended as a first-line treatment because it’s safe and simple and promotes neural plasticity in the olfactory pathway.^12^ Among the pharmaceutical interventions, corticosteroids are commonly chosen for treating COVID-19-related OD as they improve olfactory signal transmission by suppressing local inflammation, relieving mucosal edema, and mitigating the apoptosis of epithelial cells.^13^ However, studies on corticosteroids have yielded inconsistent conclusions. Vaira et al.^9^ have demonstrated marked improvement in olfactory scores in individuals treated with corticosteroids, indicating short-term benefit. In contrast, Schepens et al.^14^ and Tragoonrungsea et al.^15^ fail to observe an evident benefit in their Randomized Controlled Trials (RCTs). This difference suggests that the efficacy of corticosteroids is closely related to the timing and route of administration, as well as the duration of the patient’s disease, and remains controversial.

In recent years, as research into the mechanisms of OD deepens, various novel treatment strategies have been proposed. Among them, Platelet-Rich Plasma (PRP) injections promote the regeneration of olfactory epithelium by releasing multiple growth factors. PRP has been confirmed to improve both objective and subjective olfactory function in patients with COVID-19-related OD, though evidence from RCTs remains limited.^16^ Additionally, physical and Traditional Chinese Medicine (TCM) interventions such as acupuncture and aromatherapy have been used to promote olfactory nerve repair and have exhibited promising potential. Recently, increasing interest has been focused on local chelator therapy, which aims to reduce free calcium and metal ions in the nasal cavity. In a study by Imam et al.,^17^ intranasal administration of disodium ethylenediaminetetraacetate has been found to substantially improve the Threshold-Discrimination-Identification (TDI) scores and self-reported olfactory function in patients with post-COVID-19 OD, suggesting that chelators may represent a potentially effective therapy for the restoration of post-COVID olfactory function. This expands the utility of pharmaceutical interventions and justifies the inclusion of such novel therapies into the present study, thereby highlighting their clear comparative significance and clinical value.

Despite the constantly growing research on the treatment of COVID-19-related OD, systematic comparisons of different interventions for efficacy and safety remain scarce. Previous meta-analyses have predominantly focused on individual treatment methods, and the substantial heterogeneity in the results makes it difficult to determine the relative superiority among different therapies.^16^^,^^18^^,^^19^ A Network Meta-Analysis (NMA) integrates direct and indirect evidence and systematically compares and ranks multiple treatment methods to provide higher-level evidence-based support. Therefore, this systematic review sought to comprehensively evaluate the efficacy and safety of various pharmaceutical and non-pharmaceutical therapies for COVID-19-related OD through an NMA. It utilized the Sniffin' Sticks Test (TDI), University of Pennsylvania Smell Identification Test (UPSIT), Visual Analogue Scale (VAS), and 22-item Sino-Nasal Outcome Test (SNOT-22) as primary outcome measures to investigate the relative efficacy and advantages of the interventions. This study was expected to offer a reference basis for the individualized treatment of COVID-19-related OD.

## Methods

This study adhered to the Preferred Reporting Items for Systematic Reviews and Meta-Analyses (PRISMA) guidelines and the extension for NMA.^20^ The study protocol was registered in the International Prospective Register for Systematic Reviews (PROSPERO) (ID: CRD420251117459).

### Literature search

Two reviewers (YWL and LLX) independently searched PubMed, Embase, Cochrane Library, and Web of Science databases for articles published from the inception of the databases to July 31, 2025. The search strategy was designed by combining Medical Subject Headings (MeSH) and free-text terms, including “COVID-19”, “Coronavirus Disease 2019”, “olfaction disorders”, and “olfactory training”. To avoid omissions, we also searched the reference lists of included studies to identify additional studies. Any disagreements between reviewers were resolved through discussion or consultation with a third reviewer. Detailed search strategies for each database are presented in Table S1.

### Inclusion criteria

Inclusion criteria were established based on the Population, Intervention, Comparison, Outcomes and Study design (PICOS) framework as follows: 1) Population (P): Adult patients diagnosed with post-COVID-19 OD; 2) Intervention (I): Any active intervention for treating post-COVID-19 OD; 3) Control (C): Any placebo, sham intervention, or any other active interventions; 4) Outcome (O): Improvement in olfactory function. At least one objective or subjective assessment indicator of olfactory function must be included; 5) Study design (S): RCTs, or non-RCTs.

### Exclusion criteria

Exclusion criteria were as follows: 1) Studies enrolling participants without post-COVID-19 OD or participants under 18-years of age; 2) Studies involving interventions unrelated to the study topic; 3) Studies lacking a clearly defined control or comparison group; 4) Studies failing to report quantitative indicators for olfactory function, or studies reporting incomplete or non-poolable data; 5) Non-original studies (e.g., case reports, reviews, meta-analyses, conference abstracts, registration protocols), animal or cell studies, or mechanistic studies; 6) Duplicate publications or studies with overlapping data; 7) Articles with no full text available.

### Literature selection

All search results were imported into EndNote 21. Two authors (YWL and ZP) independently screened all potentially eligible studies after removing duplicates. Eligibility was assessed by reviewing titles and abstracts. Subsequently, the full texts of potentially relevant articles were checked based on predefined inclusion and exclusion criteria. RCTs, cohort studies, and non-RCTs designed to compare the efficacies of different therapeutic interventions for post-COVID-19 OD were included. Any discrepancies between reviewers were resolved through discussion or consultation with a third reviewer.

### Data extraction

To avoid bias from data extraction by a single person, two independent researchers (YWL and HTT) selected studies and extracted data. When consensus could not be reached between the two researchers, a third researcher (JTQ) joined the discussion to resolve discrepancies. When eligible studies were confirmed, the following data were extracted from each selected study into an Excel spreadsheet: 1) First author and country, 2) Publication year, 3) Sample size, 4) Age and gender of participants, 5) Duration of disease, 6) Comorbidities, 7) Intervention (intervention, dosage, route of administration, frequency, and duration), 8) Baseline and post-treatment outcomes (TDI and its subscales, UPSIT, VAS, and SNOT-22).

### Quality assessment

Two researchers (SMC and JTQ) assessed the quality of included studies independently using the National Institutes of Health (NIH) Quality Assessment Tool for RCTs^21^ and the Newcastle-Ottawa Scale (NOS).^22^ The NIH tool comprises 14-items that systematically assess the risk of bias across key domains: clarity of study topic, randomization process, allocation concealment, blinding (of participants, intervention providers, or outcome assessors), comparability of baseline characteristics, and completeness of follow-up (e.g., dropout rate < 20%). Each item was rated as “yes”, “no”, or “not reported”. Methodological quality was rated as “good”, “fair”, or “poor”. The NOS scale was used to score cohort studies across 3-dimensions: subject selection, comparability, and outcome assessment. The total score ranged from 0- to 9-points, with 7–9 indicating high quality, 4–6 indicating moderate quality, and < 4 indicating low quality. All studies were independently assessed for quality by two researchers. Disagreements, if any, were resolved through discussion and consultation with a third researcher (YWL).

### Statistical analysis

All outcome measures in the included studies were continuous variables. Therefore, the Standardized Mean Difference (SMD) with 95% Credible Interval (CrI) was utilized to measure effect sizes. A Bayesian NMA model was constructed using Markov Chain Monte Carlo methods. This model was iterated to estimate the relative efficacy of different treatment regimens. During model validation, 4 chains were run with a burn-in period of 10,000 iterations, followed by 50,000 sampling iterations. The thinning interval was set to 1, and the initial value was specified as 2.5, aiming to obtain posterior distributions. Heterogeneity was assessed using the mtc:anohe function in the GeMTC package. When the overall *I*^2^ < 50%, the heterogeneity among included studies within the comparison was considered acceptable, satisfying the homogeneity assumption. Consistency modeling^23^ was applied when the difference in Deviance Information Criteria (DIC) values was < 5, which indicated good consistency. The mtc.nodesplit function in the GeMTC package was employed to test the inconsistency between direct and indirect comparisons via the node splitting method. When p > 0.05, no inconsistency between direct and indirect comparisons existed, satisfying the consistency assumption. Convergence was assessed for the results by calculating the Potential Scale Reduction Factor (PSRF), with 1 as the benchmark. 1 ≤ PSRF ≤ 1.05 indicated successful convergence. A network was constructed, where the nodes of the network represented different interventions, and the lines denoted head-to-head comparisons between interventions. Cumulative probability plots were analyzed to estimate all cumulative ranking probabilities, reported as the Surface Area Under the Cumulative Ranking Curve (SUCRA) and cumulative probability rankings. A higher SUCRA value indicated a greater probability of the intervention being the most effective.^24^ Funnel plots were drawn to evaluate potential publication bias. All statistical analyses were conducted using R 4.5.0 and STATA 18.0.

## Results

### Literature search and screening process

**Fig. 1:**
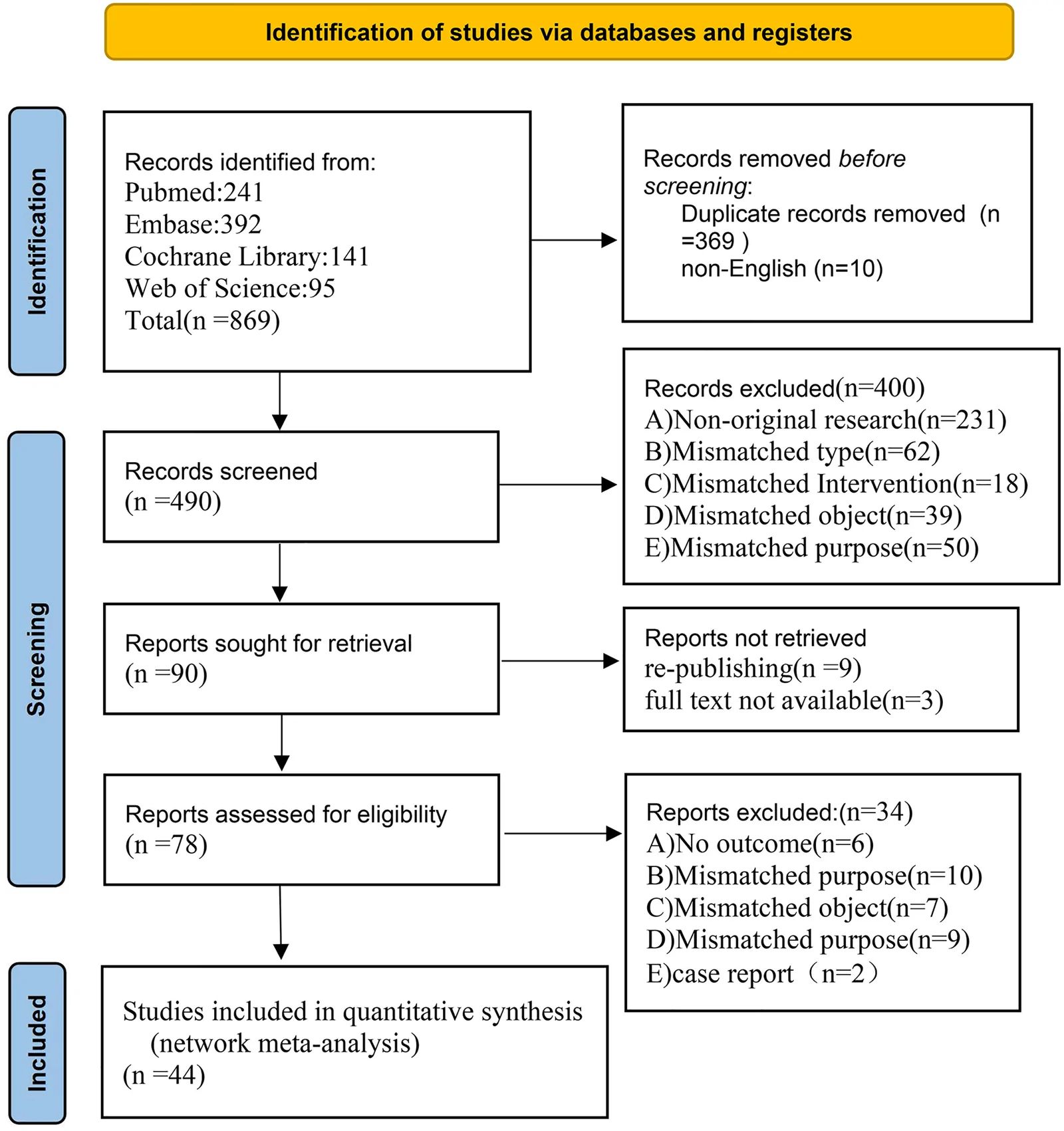
Study selection flowchart.

A total of 869 studies were retrieved from 4 databases, among which 369 duplicates were removed, and 500 studies were screened using EndNote 21. After full-text review of the 90 studies passing the initial screening, 46 studies were eliminated, including 6 for failure to report outcome measures, 9 for ineligible interventions, 10 for ineligible study objectives, 7 for ineligible study populations, 2 for being case reports, 9 for duplicate publication, and 3 for unavailability of full texts. Ultimately, 44 studies meeting the eligibility criteria were included (Fig. 1).

### Characteristics of included studies

Studies from multiple countries, including Egypt, the US, Italy, Brazil, Iran, and China, were included. Among them, 35 were RCTs, 8 were cohort studies, and 1 was a non-RCT study. All the studies were published between 2021 and 2025. Data from a total of 3,516 patients with post-COVID-19 OD were analyzed. Sample sizes ranged from 22 to 285 participants, with mean disease duration varying from 2 days to 1.8 years. The mean age of participants ranged from 30.9 to 56.1 years. The basic characteristics of the included studies are summarized in Table 1.

**Table 1:** Characteristics of included studies.

General information				Demographic information of study population					Intervention					Outcome
First author	Year	Country	Study design	Sample size	Year	Gender (M/F)	Duration of the disease (month)	Comorbidities	Name	Dose	Routine	Frequency	Duration (week)	Outcome
Figueiredo	2024	Brazil	RCT	49	38.2±11.3	10/39	/	/	OT+ALA	300 mg/pill	Po	Bid	12	VAS
				51	39.9±13.3	8/43	/	/	OT	/	/			
Filiz	2024	Canada	RCT	23	39.5±9.6	5/15	8.97±2.6	/	OT	/	/	/	12	UPSIT
				22	46.3±13.8	5/11	11.53±3.9	/	MOT	/	/	/		
				23	43.5±10.1	5/15	8.2±3.6	/	Control	/	/	/		
Masson	2025	France	RCT	49	44.8±14.4	16/33	6.8±2.7	/	OT	/	/	Bid	16	VAS
				30	38.9±13.4	3/27	7.2±1.9	/	Strengthening OT	/	/			
Tragoonrungsea	2023	Thailand	RCT	72	33.36±10.4	32/40	0.09±0.08	DM2 Allergic rhinitis8	Corticosteroid	1mg/2mL + 500 mlnacl	Intranasal irrigation	Bid	6	VAS
				70	34.97 ± 10.7	29/41	0.07±0.05	DM1 Allergic rhinitis7 Asthma 2	Control	125mlnacl				
Abdelalim	2021	Egypt	RCT	50	(20.5–38.0)	24/26	0.42±0.15	DM 8 Hypertension 6	OT + Corticosteroid	20S+100μg	Nasal spray	Qd+Bid	2	VAS
				50	(22.5–39.0)	22/28	0.4±0.17	DM 8 Hypertension 8	OT	20S	/	Bid		
Lechner	2022	UK	RCT	33	/	/	/	/	OT	/	/	/	/	UPSIT
				30	/	/	/	/	Control	/	/	/		
Abdelazim a	2022	Egypt	RCT	29	38.67±7.21	16/13	/	DM7 Asthma3 Migraine2 Hypertension6 Sinusitis3	Chelator	/	/	/	/	TDI (and subgroups)
				29	39.87±6.58	14/15	/	DM6 Asthma2 Migraine4 Hypertension5 Sinusitis2	Control	/	/	/		
Abdelazim b	2022	Egypt	RCT	32	37.98±6.27	14/18	3.22±0.14	Asthma4 DM2 Hypertension3 Migraine4 Hyperuricemia 2	Control	0.90%	Nasal spray	Tid	4	TDI (and subgroups)
				32	36.87±5.25	12/20	3.16±0.13	Asthma5 DM4 Hypertension3 Migraine5 Hyperuricemia 3	Chelator	1%				
Mogensen	2025	Denmark	RCT	25	47.28±17.29	6/19	24.25±9.04	/	OT	/	/	Bid	12	TDI (and subgroups)
				24	47.63±28.76	8/16	21±5.91	/	Control	/	/			
Abdelazim c	2022	Egypt	RCT	25	38.37±8.58	9/16	3.17±0.21	Asthma2 DM7 Hypertension5 Sinusitis7	Control	0.90%	Nasal spray	Tid	4	TDI (and subgroups)
				25	39.25±7.23	10/15	3.25±0.2	Asthma3 DM8 Hypertension3 Migraine1 Sinusitis8	Chelator	1%				
De Luca	2022	Italy	Cohort study	43	40.2±10.9	14/29	8.5±1	/	OT+PEA-LUT	6 min + 700‒70mg	SL	Tid+Qd	/	TDI
				16	37.6±8.4	10/6	8.4±1.7	/	PEA-LUT					
Steffens	2022	Belgium	Non-RCT	30	39±12	14/16	10.8±2.5	/	PRP	/	/	/	4	TDI
				26	44±11	6/20	9.7±3.4	/	Control	/	/	/		
Pendolino	2025	UK	Cohort study	12	38.35±10.48	3/9	27.1±5.0	Hypothyroidism1 Asthma 1 Others4 Allergic rhinitis 2	FSPR	/	/	/	/	TDI (and subgroup), VAS, SNOT-22
				13	43.89±19.93	5/8	28.3±9.0	Hypothyroidism 1 Asthma 1 Others3	Control	/	/	/		
Lechien	2024	France	Cohort study	81	43.5±13.4	20/61	/	Thyroid disorder 11 Depression 5 Asthma 4 Hypertension 5 DM6	PRP	0.2mL	Nasal injection	/	12	TDI (and subgroups)
				78	47.0±11.1	26/52	/	Thyroid disorder 6 Depression 8 Asthma8 Hypertension 6 DM2	Control			/		
Yaylacı	2023	Turkey	Cohort study	25	38 ± 14	10/15	5.8 ± 3.4	/	OT	/	/	/	/	TDI (and subgroups)
				18	37 ± 10	9/9	4.7 ± 3.3	/	Control	/	/	/		
Mohebbi	2024	Iran	RCT	20	31.95±8.37	9/11	/	/	TCM	20min	/	/	/	VAS
				20	30.90±7.09	7/13	/	/	Control	/	/	/		
Le Bon	2021	Belgium	Cohort study	9	42 ± 14	2/7	/	/	OT + Corticosteroid	32mg	Po	Qd	1.5	TDI
				18	44 ± 14	4/14	/	/	OT	/	/			
Khan	2023	America	RCT	61	39 ± 12	11/50	5±3	/	MOT	/	/	/	/	UPSIT
				60	43± 13	5/55	6±3	/	OT	/	/	/		
				35	43 ± 10	5/30	5±3	/	Control	/	/	/		
Abdelazim	2024	Egypt	RCT	165	43.25±7.23	85/80	6.51±0.90	Asthma 5 DM 12 Hypertension 11	Forskolin	/	Po	Tid	/	TDI (and subgroups)
				120	42.37±8.58	55/65	6.51±0.98	Asthma 6 DM 13 Hypertension 10 Migraine 1	Control	/	/	/		
Mahadev	2023	America	RCT	34	42±10.6	8/26	/	/	Gabapentin	900mg	Po	Qd	8	UPSIT
				34	44±15.5	9/25	/	/	Control					
Imam	2023	Egypt	RCT	33	38.25±7.62	/	3.27±0.21	Asthma 3 DM 8 Hypertension 8 Migraine 4	Chelator	20mg/mL	Nasal spray	/	4	TDI (and subgroups)
				33	40.37±7.58	/	3.17±0.19	Asthma 2 DM 7 Hypertension 7 Migraine 2	Control			/		
Altemani	2024	Saudi Arabia	RCT	26	41.25±7.23	13/13	3.2±0.19	Asthma 2 DM 6 Hypertension 5 Migraine 1	Chelator	1%	Nasal spray	Tid	4	TDI (and subgroups)
				26	40.37±8.58	11/15	3.17±0.18	Asthma 3 DM 8 Hypertension 3	Control					
Oliveira	2025	Brazil	RCT	28	36.4±12.7	13/15	7.9±3.7	DM 1 Hypertension2	OT+ Infrared PBMT + Corticosteroid	40S+40mg	/	Bid	5	VAS
				26	36.2±12.4	6/20	6±3.9	Asthma 1 Hypertension 2 Depression 3	OT+ Red-light + Corticosteroid	/	/			
				27	36±10.5	4/23	7.8±2.9	DM 1 Hypertension1 Depression3	OT + corticosteroid	/	/			
Armstrong	2025	America	RCT	7	52.0±22.87	2/5	/	/	OT + Corticosteroid + TCM	/	/	Bid+Qd	10	VAS, SNOT-22
				11	46.7±18.21	3/8	/	/	OT + Corticosteroid	/	/	Bid		
Pires	2022	Brazil	RCT	26	38.0 ± 9.9	7/19	/	/	OT	/	/	Bid		UPSIT, VAS
				54	36.1 ± 10.6	21/33	/	/	Strengthening OT	/	/	/		
Abdelazim	2023	Egypt	RCT	25	41.25 ± 7.23	9/16	6.0±0.2	Asthma 2 DM 6 Hypertension 5 Migraine 1	OT + Chelatingagent	1%	Nasal spray	/		TDI (and subgroups)
				25	40.37 ± 8.58	10/15	6.0 ± 0.2	Asthma 1 DM 5 Hypertension 3	OT	/	/	/		
Imam	2025	Egypt	RCT	26	38.27±6.20	12/14	0.52±0.04	Asthma 2 DM 6 Hypertension 5 Migraine 4	OT+Chelatingagent	100μL/1%	Nasal spray	Tid	8	TDI (and subgroups)
				26	37.28±6.41	12/14	0.51±0.03	Asthma 3 DM 7 Hypertension 6 Migraine 2	Control	/	/	/		
Hosseinpoor	2022	Iran	RCT	35	32.23 ± 10.02	11/24	1.92±0.57	DM 2 Hypertension 1 Cardiovascular disorder 1	Corticosteroid	0.05%	Nasal spray	Bid	4	VAS
				35	34.93 ±12.39	14/21	1.89±0.47	DM 4 Hypertension 4 Cardiovascular disorder 2	Control					
Fieux	2025	America	Cohort study	16	51.8 ±14.5	6/10	11.3±5.1	/	PRP	2mL	Nasal injection	Bid	48	UPSIT, VAS
				16	47.1±10.9	7/9	15.62	/	Control	/	/	/		
Kasiri	2021	Iran	RCT	39	35.4±9	20/19	/	Asthma 1 DM 1 Hypertension 1 Dyslipidemia 1	OT+Corticosteroid	100Âμg/ 0.05%	Nasal spray	Bid	4	VAS
				38	33.2±8.5	19/19	/	Hypertension 1 Dyslipidemia 1	OT	/	/			
Pendolino	2023	UK	Cohort study	19	43.76±18.42	8/11	8.2±5	DM 1 Hypertension 1 Hyperlipidemia 1 Hypothyroidism 1	OT+Corticosteroid	/	/	/	24	TDI (and subgroups), VAS, SNOT-22
				16	46.38±19.51	5/11	9.8±7.9	Hypertension 3 Hyperlipidemia 2	OT	/	/	/		
				9	31.63±6.12	3/6	9.1±5.6	/	Control	/	/	/		
Berube	2023	Canada	RCT	25	44.9±7.4	9/16	9.1±3.6	/	OT	/	/	Bid	12	UPSIT, VAS
				25	44.5±10.1	8/17	8.0±3.7	/	Control	/	/			
Lerner	2022	America	RCT	57	41.5±14.6	11/46	6.5±3.5	Ageusia:41 History of Facial Trauma:2 Chronic Rhinosinusitis:1 Allergic Rhinitis:3 Neurodegenerative Disease:1	Omega-3	1000mg	Po	/	6	UPSIT
				60	40.7±12.7	14/46	6.8±3.2	Ageusia:48 Chronic Rhinosinusitis:1 Allergic Rhinitis:6	Control			/		
Hernandez	2022	Germany	RCT	29	46.1±15	16/13	9.2±7.2	/	OT+Omega-3	1g	Po	Bid	12	TDI (and subgroups)
				29	53±16.5	9/20	13.3±25.8	/	OT	/	/	/		
Gellrich	2024	Germany	RCT	34	53.6±11	21/13	21.6±17	/	OT+PEA-LUT	700 mg + 70 mg	Po	Tid+Qd	13	TDI (and subgroups)
				16	45.5±13	7/9	12.8±5	/	OT	/	/	/		
Stadio	2023	Italy	RCT	94	36.7±11.8	45/49	8.8±3.68	Hypertension1 Allergy 5 Thyroid Disorders 6 Psychiatric disturbances 2	OT+PEA-LUT	700 mg + 70 mg	Po	Qd	12	I
				36	50.5±12.7	15/21	8.5±1.97	Hypertension2 Allergy 2 Thyroid Disorders 2 Psychiatric disturbances 6	OT	/	/	/		
Cantone	2024	Italy	RCT	23	52.1 ± 11.8	10/13	8.1 ± 1.8	/	OT	/	/	Qd	/	TDI
				17	44.8 ± 11.81	6/11	10 ± 4.4	/	OT+PEA-LUT	700 mg + 70 mg	/			
				21	35.9 ± 12.3	12/9	10.2 ± 4.13	/	OT+ALA	800mg	/			
				28	42 ± 10.29	10/18	15.3 ± 6.6	/	OT+PEA-LUT+ALA	/	/			
Chung	2023	China	RCT	9	34.89±14.87	4/5	4.99±0.96	/	OT+VitA	25,000 IU	Po	Tid	4	UPSIT
				8	47.35±16.98	2/6	5.1±1.8	/	OT	/	/	/		
				5	56.06±11.06	2/3	5.7±3.0	/	Control	/	/	/		
Genetzaki	2021	Greece	Cohort study	78	/	/	/	/	OT + Corticosteroid	40mg	Po	Qd	2	TDI
				53	/	/	/	/	OT	/	/			
Schepens	2022	Netherlands	RCT	58	50.93±12.77	24/34	/	/	OT + Corticosteroid	40mg	Po	Qd	12	TDI (and subgroups), SNOT-22, VAS
				57	46.88±11.79	18/39	/	/	OT	/	/			
Vestito	2025	Italy	RCT	35	47.17±12.96	14/21	18.06±7.15	/	OT+A-tDCS	/	/	Qd	2	TDI
				17	44.47±10.70	6/11	16.29±6.59	/	OT	/	/			
Mohamad	2022	Egypt	RCT	60	52.65±7.67	10/50	0.36±0.11	/	Corticosteroid + Antihistamine	50µg+125µg	Nasal spray	Tid	/	D
				60	51.90±7.25	9/51	0.36±0.1	/	Corticosteroid	/	/			
				60	50.85±6.74	6/54	0.36±0.1	/	Antihistamine	/	/			
				60	51.98±6.95	46/14	0.34±0.1	/	Control	/	/			
Algarni	2025	Saudi Arabia	RCT	33	46.25±7.88	18/15	6.2±1.1	Asthma 4 DM 8 Hypertension 8 Migraine 1	Chelator	/	Nasal spray	/	/	TDI (only subgroup)
				33	45.37±8.50	12/25	6.1±1.2	Asthma 3 DM 8 Hypertension 9	Control	/		/		
Duffy	2024	America	RCT	41	50±15	14/27	18±7	/	PRP	/	/	/	12	UPSIT
				42	51±15	10/32	19±6	/	Control	/	/	/		

Note: po, per os; Qd, Quaque die; Bid, Bis in die; Tid, Ter in die; SL, Sublingual; M, Male; F, Female; TDI, Sniffin' Sticks Test; UPSIT, University of Pennsylvania Smell Identification Test; VAS, Visual Analogue Scale; SNOT-22, 22-item Sino-Nasal Outcome Test; D, Discrimination; I, Identification; OT, Olfactory Training; MOT, Multisensory Olfactory Training; PEA-LUT, Palmitoylethanolamide and Luteolin; FSPR, Functional Septorhinoplasty; TCM, External Treatment with traditional Chinese Medicine; Infrared PBMT, Infrared Photobiomodulation Therapy; A-tDCS, Anodal Transcranial Direct Current Stimulation; PRP, Platelet-Rich Plasma; ALA, Alpha-Lipoic Acid; VitA, Vitamin A; DM, Diabetes Mellitus; /, Not mentioned.

### Quality assessment results

Quality assessment of the included RCTs utilizing the NIH scale revealed that 14 studies^14^^,^^15^^,^^17^^,^^25^, ^26^, ^27^, ^28^, ^29^, ^30^, ^31^, ^32^, ^33^, ^34^, ^35^ were rated as having good quality, 20^36^, ^37^, ^38^, ^39^, ^40^, ^41^, ^42^, ^43^, ^44^, ^45^, ^46^, ^47^, ^48^, ^49^, ^50^, ^51^, ^52^, ^53^, ^54^, ^55^ were rated as having fair quality, and 2^56^^,^^57^ were rated as having poor quality. The low quality of the RCTs primarily stemmed from inadequate reporting of random sequence generation and allocation concealment. Quality assessment of the included cohort studies utilizing the NOS scale revealed that 6 studies^58^, ^59^, ^60^, ^61^, ^62^, ^63^ were rated as having good quality and 2 studies^64^^,^^65^ were rated as having fair quality. The low quality of cohort studies primarily stemmed from inadequate selection of the non-exposed groups and limited control of confounders. The comprehensive assessment results for risk of bias are presented in Table S2.

## NMA results

### Network diagram

**Fig. 2:**
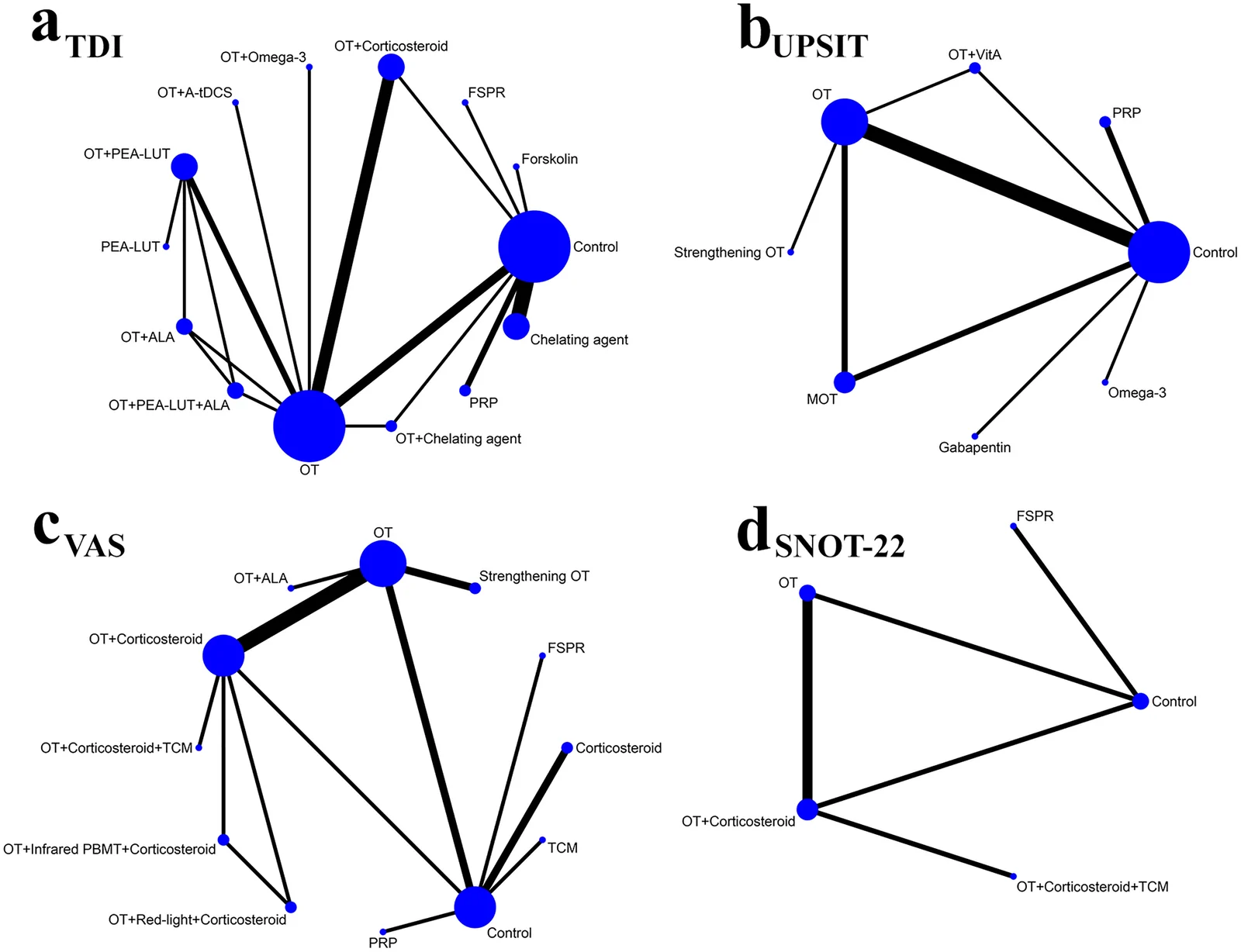
Network diagrams of different treatment methods in patients with COVID-related olfactory dysfunction.

In the network diagram, each dot represented an intervention. The size of the dot was positively correlated to the number of studies involving that intervention, namely, a larger dot indicated a greater number of included studies. A line connecting two dots indicated direct comparisons between the two interventions. The thickness of the line represented the number of studies comparing the two interventions, namely, a thicker line denoted a greater number of comparative studies, as shown in Fig. 2. All PSRF values were 1, indicating successful model convergence for all outcomes. Model inconsistency was assessed using the DIC and node splitting analysis. All differences in DIC values were below 5, indicating overall model consistency across the outcomes. Node splitting analysis was performed for each outcome with a closed loop. Results showed p > 0.05 for all outcome measures, indicating no local inconsistency (Fig. S1).

## NMA results

### Objective olfactory function assessment

#### TDI

**Fig. 3:**
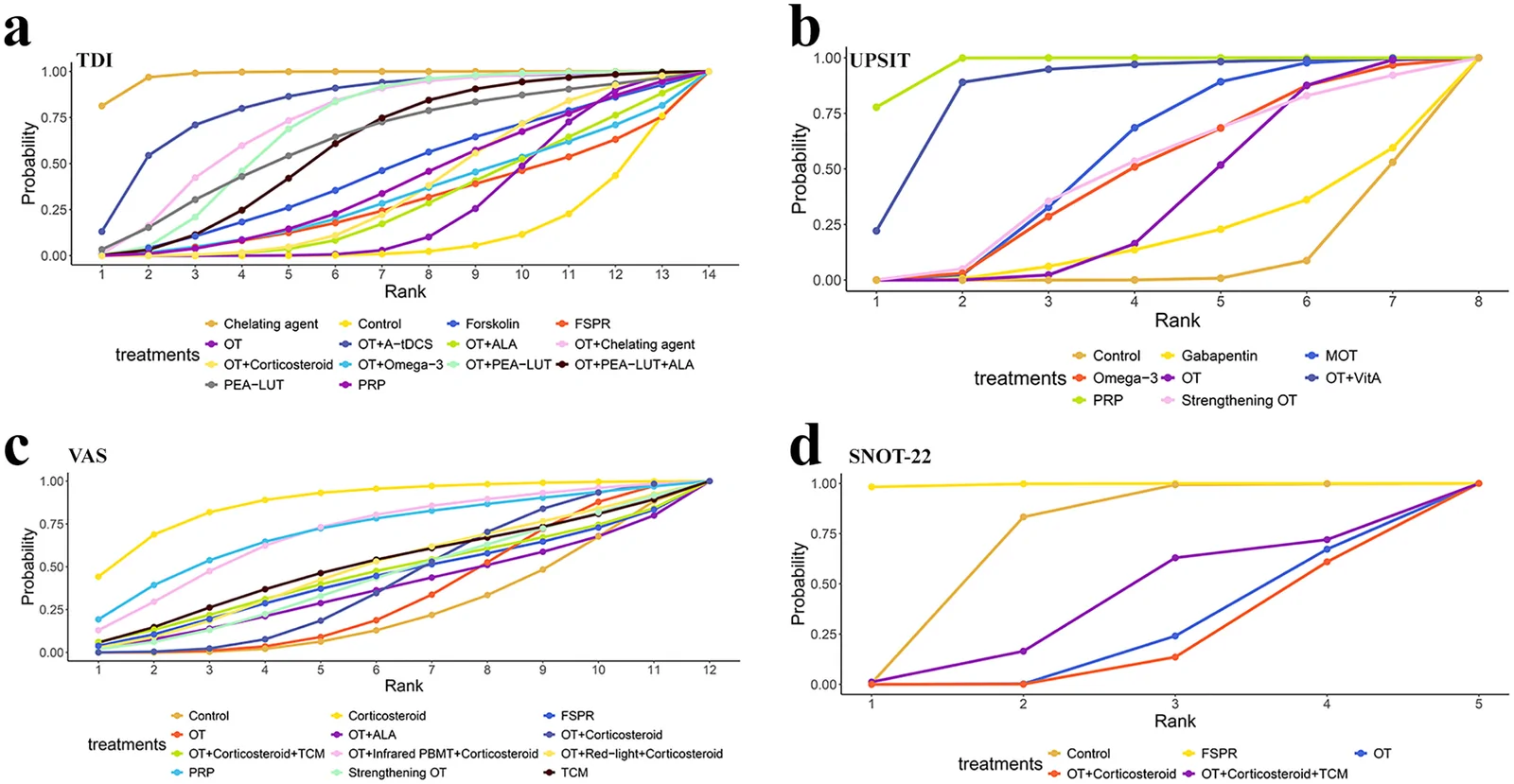
SUCRA for different treatment methods in patients with COVID-related olfactory dysfunction.

Among the included studies, 22 reported the effects of various interventions on total olfactory function scores in patients with post-COVID-19 OD. These studies enrolled a total of 1,713 patients and involved 14 different interventions, primarily including OT, chelators, and OT + corticosteroids. Fig. 2a displays the pairwise comparison network for all studies. Global inconsistency testing via NMA indicated no significant inconsistency across the network (p > 0.05) (Fig. S2a). According to heterogeneity analysis, low heterogeneity existed among most studies, but high heterogeneity was observed among some studies (Fig. S3a). Table 2a presents the league table displaying pairwise comparison results between interventions and their respective CrI. The results indicated that compared to placebo control, chelators (SMD = 3.61, 95% CrI: 2.77, 4.55), OT + Anodal transcranial Direct Current Stimulation (A-tDCS) (SMD = 2.45, 95% CrI: 0.32, 4.57), and OT + chelators (SMD = 1.83, 95% CrI: 0.43, 3.27) improved total olfactory function scores in patients with post-COVID-19 OD. Fig. 3a ranks the interventions based on cumulative probability plots and SUCRA values. The line chart of SUCRA probability ranking suggested that chelators (SUCRA = 98.2%) ranked highest in the current network, suggesting the greatest likelihood of being the optimal intervention, followed by OT + A-tDCS (SUCRA = 85.8%), and OT + Chelators (SUCRA = 81.5%).

**Table 2:** League table of the efficacy of different treatment methods in patients with COVID-related olfactory dysfunction.

**(a)** League table of TDI of different treatment methods in network meta-analysis.													
**Chelators**													
1.16 (-1.07, 3.51)	**OT+A-tDCS**												
**1.77 (0.14, 3.5)**	0.62 (-1.77, 2.97)	**OT + Chelators**											
**1.9 7(0.37, 3.7)**	0.82 (-1.37, 2.99)	0.2 (-1.57, 1.99)	**OT + PEA-LUT**										
2.08 (-0.35, 4.64)	0.92 (-1.95, 3.78)	0.31 (-2.26, 2.89)	0.11 (-1.75, 1.96)	**PEA-LUT**									
**2.27 (0.54, 4.16)**	1.11 (-1.16, 3.39)	0.5 (-1.39, 2.41)	0.3 (-0.97, 1.57)	0.19 (-2.05, 2.44)	**OT + PEA-LUT + ALA**								
**2.7 (0.78, 4.79)**	1.56 (-1.22, 4.32)	0.95 (-1.33, 3.23)	0.74 (-1.56, 3.02)	0.64 (-2.31, 3.56)	0.45 (-1.94, 2.84)	**Forskolin**							
**2.9 (1.39, 4.53)**	1.75 (-0.75, 4.22)	1.13 (-0.79, 3.07)	0.93 (-1.02, 2.86)	0.83 (-1.84, 3.5)	0.64 (-1.42, 2.66)	0.19 (-2.03,2.39)	**PRP**						
**2.96 (1.59, 4.45)**	1.8(-0.3,3.87)	1.19 (-0.41, 2.8)	0.99 (-0.39, 2.37)	0.88 (-1.43, 3.21)	0.69 (-0.84, 2.22)	0.25 (-1.86, 2.36)	0.06 (-1.66, 1.77)	**OT + Corticosteroid**					
**3.1 (0.93, 5.42)**	1.95(-0.71,4.59)	1.33 (-0.98, 3.67)	1.14 (-1.02, 3.28)	1.03 (-1.79, 3.85)	0.84 (-1.41, 3.08)	0.39 (-2.35, 3.13)	0.2 (-2.22, 2.66)	0.14 (-1.89, 2.16)	**OT + Omega-3**				
**3.14 (1.41, 4.99)**	1.98 (-0.29, 4.26)	1.37 (-0.5, 3.29)	1.17 (-0.09, 2.44)	1.06 (-1.17, 3.32)	0.87 (-0.45, 2.19)	0.43 (-1.95, 2.81)	0.24 (-1.79, 2.27)	0.18 (-1.34, 1.71)	0.04 (-2.2, 2.27)	**OT+ALA**			
**3.27 (1.2, 5.47)**	2.11 (-0.75, 4.97)	1.49 (-0.88, 3.92)	1.29 (-1.09, 3.69)	1.18 (-1.82, 4.21)	1 (-1.48, 3.47)	0.55 (-2.07, 3.2)	0.36 (-1.95, 2.69)	0.3 (-1.92, 2.54)	0.17(-2.65,2.98)	0.12 (-2.33, 2.58)	**FSPR**		
**3.19 (1.97, 4.53)**	**2.03 (0.12, 3.93)**	**1.42 (0.02, 2.83)**	**1.21 (0.14, 2.3)**	1.11 (-1.03, 3.25)	0.92 (-0.36, 2.18)	0.47 (-1.54, 2.5)	0.28 (-1.3, 1.88)	0.23(-0.63,1.08)	0.08 (-1.76, 1.92)	0.05 (-1.22, 1.31)	-0.08 (-2.22, 2.05)	**OT**	
**3.61 (2.77, 4.55)**	**2.45 (0.32, 4.57)**	**1.83 (0.43, 3.27)**	**1.63 (0.22, 3.05)**	1.53 (-0.8, 3.86)	1.34 (-0.24, 2.89)	0.89 (-0.91, 2.68)	0.7 (-0.59, 2)	0.64(-0.47,1.76)	0.5 (-1.57, 2.57)	0.46 (-1.1, 2.02)	0.34 (-1.6, 2.26)	0.42 (-0.5, 1.33)	**Control**
**(b)** League table of UPSIT of different treatment methods in network meta-analysis.													
**PRP**													
0.33 (-0.53, 1.2)	**OT+VitA**												
**0.98 (0.5, 1.47)**	0.65 (-0.14, 1.43)	**MOT**											
**1.01 (0.35, 1.67)**	0.68 (-0.21, 1.57)	0.03 (-0.5, 0.56)	**Strengthening OT**										
**1.03 (0.49, 1.58)**	0.7 (-0.14, 1.53)	0.05 (-0.4, 0.49)	0.02 (-0.61, 0.65)	**Omega-3**									
**1.11 (0.65, 1.57)**	**0.78 (0.02, 1.54)**	0.13 (-0.12, 0.38)	0.1 (-0.37, 0.57)	0.08 (-0.34, 0.51)	**OT**								
**1.29 (0.66, 1.91)**	**0.96 (0.06, 1.85)**	0.31 (-0.23, 0.85)	0.28 (-0.42, 0.98)	0.26 (-0.34, 0.86)	0.18 (-0.34, 0.7)	**Gabapentin**							
**1.33 (0.92, 1.74)**	**1 (0.24, 1.76)**	**0.35 (0.09, 0.61)**	0.32 (-0.19, 0.84)	0.3 (-0.06, 0.66)	0.22 (0.01, 0.43)	0.04 (-0.43, 0.51)	**Control**						
**(c)** League table of SNOT-22 of different treatment methods in network meta-analysis.													
**FSPR**													
**-1.06 (-1.91, -0.21)**	**Control**												
**-1.62 (-3.04, -0.21)**	-0.56 (-1.69, 0.57)	**OT + Corticosteroid + TCM**											
**-1.83 (-2.88, -0.79)**	**-0.77 (-1.37, -0.16)**	-0.22 (-1.21, 0.79)	**OT**										
**-1.86 (-2.9, -0.82)**	**-0.8 (-1.4, -0.19)**	-0.24 (-1.19, 0.71)	-0.03 (-0.34, 0.28)	**OT + Corticosteroid**									

Note: OT, Olfactory Training; A-tDCS, Anodal Transcranial Direct Current Stimulation; PEA-LUT, Palmitoylethanolamide and Luteolin; PRP, Platelet-Rich Plasma; ALA, Alpha-Lipoic Acid; FSPR, Functional Septorhinoplasty.

Note: PRP, Platelet-Rich Plasma; OT, Olfactory Training; VitA, Vitamin A; MOT, Multisensory Olfactory Training.

Note: FSPR, Functional Septorhinoplasty; OT, Olfactory Training; TCM, External Treatment with Traditional Chinese Medicine.

#### TDI subscales

A total of 17, 18, and 18 studies reported the effects of different interventions on the three dimensions of TDI, Threshold (T), Discrimination (D), and Identification (I), respectively, in patients with post-COVID-19 OD. Global inconsistency testing via NMA indicated no significant inconsistency across the network (p > 0.05) (Fig. S2b‒2d). According to heterogeneity analysis, low heterogeneity existed among most studies, but high heterogeneity was observed among some studies (Fig. S3b‒3d). Tables S3a‒3c presents the league tables displaying pairwise comparison results between interventions and their respective CrI. Compared with placebo control, chelators improved the olfactory function in patients with post-COVID-19 OD, with SMDs of 2.89 (95% CrI: 1.47, 4.45), 1.93 (95% CrI: 1.38, 2.5), and 2.64 (95% CrI: 2.02, 3.29), respectively, as depicted in Figure S4a. In the ranking of interventions based on cumulative probability plots and SUCRA values, chelators exhibited the highest probability of being the optimal intervention for the three dimensions, T, D, and I, with SUCRA values of 90.6%, 95.5%, and 99%, respectively, as shown in Figure S4b. Table S4 provides a brief overview of the specific chelators included in the studies and their main results.

#### UPSIT

A total of 10 studies reported the effects of various interventions on the olfactory identification ability of patients with post-COVID-19 OD. These studies involved 739 patients across 8 distinct interventions, primarily including OT, OT + VitA, and Multisensory Olfactory Training (MOT). Fig. 2b displays the pairwise comparison network of all studies. Global inconsistency testing via NMA indicated no significant inconsistency across the network (p > 0.05) (Fig. S2e). Heterogeneity testing revealed substantial heterogeneity among studies (Fig. S3e). Table 2b presents the league table displaying pairwise comparison results and their respective CrI. The results suggested that compared to placebo control, PRP (SMD = 1.33, 95% CrI: 0.92, 1.74), OT + VitA (SMD = 1, 95% CrI: 0.24, 1.76), and MOT (SMD = 0.35, 95% CrI: 0.09, 0.61) improved the olfactory identification ability of patients with post-COVID-19 OD. Fig. 3b ranks the interventions based on cumulative probability plots and SUCRA values. The line chart of SUCRA probability ranking indicated that PRP (SUCRA = 96.8%) had the highest probability of being the optimal intervention, followed by OT + VitA (SUCRA = 85.9%) and MOT (SUCRA = 55.6%).

### Subjective olfactory function assessment

#### VAS

A total of 15 studies reported the effects of various interventions on subjective olfactory function in patients with post-COVID-19 OD. These studies involved 1,053 patients across 12 different interventions, primarily including OT, OT + corticosteroids, and strengthening OT. Fig. 2c displays the pairwise comparison network of all studies. Global inconsistency testing via NMA indicated no pronounced inconsistency across the entire network (p > 0.05) (Fig. S2f). Heterogeneity testing revealed low heterogeneity among most studies, with high heterogeneity observed among some studies (Fig. S3f). Table S3d presents the league table displaying pairwise comparison results and their respective CrI. The results indicated that compared with placebo controls, corticosteroids (SMD = 1.99, 95% CrI: 0.52, 3.48) improved subjective olfactory function in patients with post-COVID-19 OD. Fig. 3c ranks the interventions based on cumulative probability plots and SUCRA values. The line chart of SUCRA probability ranking indicated that corticosteroids (SUCRA = 87.9%) had the highest probability of being the optimal intervention, followed by PRP (SUCRA = 70.5%) and OT + infrared Photobiomodulation Therapy (PBMT) + corticosteroids (SUCRA = 70.2%).

### OD-related quality of life

#### SNOT-22

The effects of different interventions on the quality of life in patients with post-COVID-19 OD were reported in 4 studies. These studies involved 202 patients across 5 distinct interventions, primarily including OT, OT + corticosteroids, and Functional Septorhinoplasty (FSPR). Fig. 2d displays the pairwise comparison network for all studies. Global inconsistency testing via NMA indicated no significant inconsistency across the network (p > 0.05) (Fig. S2g). Heterogeneity testing revealed low heterogeneity among studies (Fig. S3g). Table 2c presents the league table displaying pairwise comparison results of interventions and their respective CrI. The results indicated that FSPR (SMD = -1.06, 95% CrI: -1.91, -0.21) improved the quality of life in patients with post-COVID-19 OD compared to placebo control. Fig. 3d ranks the interventions based on cumulative probability plots and SUCRA values. The line chart of SUCRA probability ranking indicated that FSPR (SUCRA = 99.5%) had the highest probability of being the optimal intervention, followed by control (SUCRA = 70.8%) and OT + corticosteroids + external treatment with TCM (SUCRA = 38.4%).

### Sensitivity analysis

To assess the potential impact of different study designs on the results of the main analysis, we excluded non-RCT studies and retained only RCTs to conduct the sensitivity analysis. Regarding TDI-related outcomes, a total of 14 RCTs were included. The results showed that, compared with the placebo control, chelators improved patients’ total olfactory function scores (SMD = 3.72, 95% CrI: 2.42, 5.16). The SUCRA plot showed that chelators (SUCRA = 92.3%) were the most likely to be the optimal intervention. A total of 13, 14, and 14 RCTs reported the effects of different interventions on T, D, and I, respectively. The results showed that, compared with the placebo control, chelators demonstrated improvements across all three dimensions: T (SMD = 2.97, 95% CrI: 0.66, 5.36), D (SMD = 1.93, 95% CrI: 1.3, 2.6), and I (SMD = 2.64, 95% CrI: 1.87, 3.46). The SUCRA plot indicated that chelators were the most likely to be the optimal intervention for T (SUCRA = 80.4%), D (SUCRA = 92.8%), and I (SUCRA = 94.1%).

For the UPSIT outcome, a total of 9 RCTs were included. The results showed that, compared with the placebo control, PRP (SMD = 1.47, 95% CrI: 0.98, 1.95), OT + VitA (SMD = 1, 95% CrI: 0.24, 1.76), and MOT (SMD = 0.35, 95% CrI: 0.09, 0.61) improved patients’ UPSIT scores. The SUCRA plot showed that PRP (SUCRA = 97.8%) was the most likely to be the optimal intervention, followed by OT+VitA (SUCRA = 84.8%) and MOT (SUCRA = 55.7%). For the VAS outcome, a total of 12 RCTs were included. The results showed that none of the interventions demonstrated statistically significant differences compared to the placebo control. The SUCRA plot indicated that corticosteroids (SUCRA = 78%) were the most likely to be the optimal intervention, followed by OT + infrared PBMT + corticosteroids (SUCRA = 68.8%) and OT + red light + corticosteroids (SUCRA = 51.8%). For the SNOT-22 outcome, since only two RCTs were included, a sensitivity analysis including only RCTs was not conducted. Table S5 and Figure S5 show the league table and SUCRA plot.

This study grouped various chemical chelators into the same intervention category. However, the pharmacological characteristics and potential efficacy of specific drugs are not entirely the same. To further evaluate the impact of different chemical chelators on primary outcomes, sensitivity analyses were conducted by sequentially excluding studies on specific chelators. The results showed that after sequential exclusion of studies, the effect size of chelators for the TDI total score remained overall stable compared with the placebo control, ranging between SMD = 2.99 (95% CrI: 2.38, 3.62) and SMD = 3.83 (95% CrI: 2.86, 4.93). This indicated good robustness of the results (Table S6).

### Publication bias

Comparison-adjusted funnel plots were drawn to evaluate the publication bias. All plots were symmetrical, indicating no evident publication bias (Fig. S6).

## Discussion

This study represents the first to systematically evaluate multiple interventions for post-COVID-19 OD via an NMA. According to the results, Chemical chelators demonstrated improvements in TDI and its three subscales, while PRP, OT + VitA, and MOT performed well in UPSIT outcomes. In contrast, evidence regarding VAS and SNOT-22 outcomes is relatively limited, and the results should be interpreted with caution. A sensitivity analysis including only RCTs showed that the primary outcomes for the TDI, its three subscales, and the UPSIT were consistent with the overall direction of the main analysis. However, no statistically significant differences were observed for VAS outcomes, suggesting that some subjective outcomes may be more likely to be influenced by study design, sample size, and assessment methods.

Regarding objective olfactory function, chelators demonstrated improvement in both the TDI total score and its three subscales. Previous studies have suggested that COVID-19-related OD is associated not only with damage to the olfactory epithelium and local inflammation but also with an imbalance in the local ionic environment of the nasal cavity, particularly elevated levels of free calcium. Excessive free calcium can inhibit olfactory signal transduction, which is detrimental to the recovery of olfactory function.^26^^,^^66^, ^67^, ^68^ Research has found that chelators are believed to mitigate the inhibition of olfactory signal transduction by reducing local free calcium concentrations, thereby improving olfactory function.^27^, ^28^, ^29^ The findings of Altemani et al.^28^ indicate that sodium phytate can improve patients’ olfactory function by regulating the local calcium ion environment of the nasal cavity. Another study suggests that the combination of chelators and OT may provide additional benefit by improving the local microenvironment and promoting neuroplasticity.^29^ It should be noted that our study included chelators as a single intervention category in the NMA, primarily based on their consistent effect direction.^27^, ^28^, ^29^ However, this node encompasses a variety of different molecules, such as Ethylene Diamine Tetraacetic Acid (EDTA), Nitrilotriacetic Acid Trisodium salt (NTA), Pentasodium Diethylenetriamine Pentaacetate (DTPA), and Glutamate Diacetate (GLD), among others. The pharmacological characteristics and potential efficacy of these agents are not entirely consistent.^17^^,^^25^, ^26^, ^27^, ^28^, ^29^^,^^35^^,^^42^ Different chelators show varying magnitudes of improvement in TDI and the reduction in calcium concentration in the nasal cavity. Some agents exhibit substantial improvements in TDI, alongside more significant decreases in calcium concentration in the nasal cavity.^17^^,^^25^^,^^27^^,^^29^ In contrast, other agents show some calcium-lowering effects but relatively smaller improvements in TDI.^26^^,^^28^^,^^42^ One study did not fully report changes in TDI.^35^ Due to the current lack of direct head-to-head comparisons between different chelators, it is impossible to further determine whether there are differences in therapeutic efficacy or potency among specific chelators in our study. Therefore, the results of this study merely indicate that this category of interventions as a whole shows certain potential. It cannot be inferred that all specific chelators have the same therapeutic efficacy or potency. Additionally, our study found that OT + A-tDCS also significantly improved overall olfactory function. A-tDCS enhances neuronal excitability and synaptic plasticity through low-intensity direct current stimulation, thereby promoting functional remodeling of the olfactory cortex.^69^ A study by Vestito et al.^33^ also demonstrates that OT + A-tDCS can improve TDI scores in patients with persistent COVID-19-related olfactory loss. In contrast, the degree of improvement with OT alone or OT combined with corticosteroids is limited, and their efficacy may still be influenced by factors such as training intensity, treatment duration, and differences in the disease course of patients.^14^^,^^70^ Furthermore, a sensitivity analysis including only RCTs showed that the effect direction of chelators for the aforementioned outcomes remained generally consistent, indicating the robustness of the results.

Regarding the olfactory identification ability outcome (UPSIT), PRP, OT + VitA, and MOT all demonstrated some benefit. The results remained generally stable in the analysis including only RCTs, suggesting their potential benefit in improving olfactory identification ability. A previous study has suggested that COVID-19-related OD primarily involves damage to the peripheral olfactory system, including inflammation of the olfactory epithelium, damage to supporting cells, and disruption of the mucosal barrier, which in turn affects the normal activation of olfactory receptor neurons.^71^ PRP is rich in various bioactive factors that can promote tissue repair, epithelial regeneration, and neural plasticity,^56^^,^^72^ and improve olfactory function by reducing local inflammation and promoting the regeneration of olfactory nerves and the olfactory epithelium.^73^^,^^74^ A study by Lechien et al.^75^ also demonstrates that PRP injections significantly reduce inflammatory responses in the olfactory epithelium and are well-tolerated, directly supporting the inhibitory effect of PRP on olfactory epithelial inflammation. OT + VitA also exhibited relatively stable improvements in olfactory identification ability. A study by Chung et al.^54^ suggests that oral VitA combined with OT can significantly improve UPSIT scores in patients with long COVID. VitA activates the retinoic acid signaling pathway, promotes the differentiation of olfactory epithelial stem cells and the regeneration of olfactory receptor neurons, and improves the regenerative capacity of the olfactory nerve, thereby enhancing the olfactory identification ability.^76^^,^^77^ Additionally, MOT also demonstrated some therapeutic benefit in our study, suggesting that cross-sensory input may promote the remodeling of olfactory identification function by enhancing cross-modal network connections in the olfactory cortex.^78^ In contrast, gabapentin did not show a clear benefit in this study. However, as this finding is currently based on a single study, the evidence base is limited, and its true efficacy cannot be definitively determined.

Regarding subjective olfactory function scores, corticosteroids demonstrated a certain advantage over the control. Corticosteroids can improve the local nasal microenvironment by inhibiting the release of local inflammatory factors, alleviating mucosal edema, and reducing epithelial cell damage, thereby optimizing patients’ subjective experience.^30^ However, existing research findings regarding their efficacy are inconsistent. A study by Saussez^13^ has noted that the use of corticosteroids yields short-term benefit, whereas Tragoonrungsea et al.^15^ have noted that although early application of glucocorticoid nasal irrigation reduces the risk of nasal dryness and discomfort, it does not accelerate olfactory recovery. These discrepancies may be related to the disease duration of patients, method of administration, dosage, and treatment duration.^75^ In the sensitivity analysis of this study, which included only RCTs, no statistically significant differences were observed among the various interventions regarding VAS outcomes. This finding may be attributed to reduced statistical power due to the smaller sample size of the included studies. Additionally, as a subjective outcome measure, VAS may also be influenced by variations in patient perception, assessment methods, and follow-up time. Therefore, the results regarding the potential role of corticosteroids in the improvement of subjective olfaction should be interpreted with caution. Furthermore, OT + ALA did not demonstrate additional efficacy for this outcome measure, which is consistent with the findings of a previous study.^36^

For the SNOT-22 outcome, FSPR showed some benefit. A previous study has shown that functional nasal surgery can improve postoperative olfactory function, nasal airflow, and nasal-related quality of life.^79^ A study by Pendolino et al.^59^ has found that FSPR can improve olfactory scores in patients with long-COVID-related OD, and its efficacy is positively correlated with increased postoperative nasal airflow. Furthermore, a neuroimaging study has suggested that FSPR not only improves nasal structure and airflow distribution but may also induce changes in the structural and functional plasticity of the central olfactory network, thereby promoting olfactory perception and integration.^80^ However, this finding is currently supported by only a very small number of studies, and a sensitivity analysis including only RCTs is not conducted. Therefore, this finding should be interpreted with caution. In contrast, OT alone and OT combined with corticosteroids did not demonstrate a clear benefit for this outcome. A study has suggested that this may be related to insufficient training duration, stimulation frequency, or variety of odors.^70^

### Limitations

Although this study systematically integrated multiple existing studies on the treatment of COVID-19-related OD, several limitations remain. For instance, the main analysis included data from both RCTs and non-RCTs. Variations among studies in terms of randomization methods, allocation concealment, blinding, and control of confounders may increase methodological heterogeneity and affect the pooled effect estimates. Further sensitivity analyses including only RCTs were conducted. While the overall effect direction for the primary outcomes was largely consistent with the main analysis, the precision of effect estimates for some outcomes decreased, suggesting that the inclusion of studies with different designs may affect the stability of the results. Therefore, the findings should be interpreted with caution. Secondly, the included studies employed inconsistent diagnostic criteria for OD, and the baseline severity of OD varies. This study failed to conduct subgroup analyses based on different diagnostic criteria and severity degrees within the network, potentially affecting the interpretation of effect sizes. Third, the methods for olfactory function assessment varied, and differences existed in the sensitivity and reliability among the objective and subjective scales utilized across studies. Although the scales were standardized, this may still introduce bias in effect size estimates. Fourth, the limited number of studies included for some interventions resulted in relatively weak indirect evidence chains within the network structure. The lack of direct comparisons and the weak indirect evidence chains limit the stability and accuracy of the effect estimates and ranking results. Furthermore, in this study, chelators are grouped into a single category of intervention for analysis, which actually encompasses various distinct molecules, such as EDTA, NTA, DTPA, and GLD. Given the lack of direct comparisons between these agents and the fact that their pharmacological characteristics and potential efficacy may not be entirely the same, grouping them under a single node may introduce heterogeneity and, to some extent, overestimate their overall efficacy. Furthermore, the potential impact of publication bias cannot be entirely ruled out. Although funnel plots are overall symmetrical, the effects of some interventions may be overestimated, given that negative results may be underreported, and most trials have small sample sizes. Additionally, substantial heterogeneity exists in study designs and methodologies. The studies exhibit considerable heterogeneity in randomization methods, blinding, and follow-up duration. Some studies lack rigorous allocation concealment and blinding designs, which may introduce selection and detection biases. Therefore, our findings remain to be validated in larger-scale, multicenter, high-quality RCTs.

### Clinical implications and future directions

This study provides a systematic, evidence-based reference for treatment strategies for COVID-19-related OD through a Bayesian NMA. The results suggest that chelators and some combination therapies show some potential for restoring objective olfactory function. However, given that some interventions are supported by only a small number of studies and that some comparisons rely on indirect evidence, the findings should be interpreted with caution. Therefore, this study may only serve as a reference for individualized interventions for persistent OD following COVID-19, rather than providing definitive conclusions. Future multicenter, large-scale, high-quality RCTs should be conducted, particularly for treatments with currently limited evidence, such as gabapentin, omega-3, and some combination therapies, to mitigate the impact of isolated nodes in the network and sparse comparisons on the stability of the results. Second, given that the chelators node in this NMA encompasses multiple distinct molecules, direct comparisons between specific chelators should be conducted to clarify differences in efficacy and potency among these agents. Additionally, researchers are encouraged to publish negative results or data showing no statistical significance to reduce publication bias. Furthermore, future studies should adopt standardized study designs, clearly define randomization strategies, and strictly implement allocation concealment and standardized follow-up durations to reduce methodological heterogeneity. Finally, standardized criteria for olfactory function assessment and efficacy evaluation should be established. Objective measures such as the Sniffin’ Sticks TDI or UPSIT are recommended as primary outcomes to precisely quantify the degree of olfactory function recovery, thereby enhancing the comparability of results across studies.

## Conclusion

The results of this study indicated that different treatment strategies were differently effective in recovering olfactory function and improving quality of life. Chelators and some combination therapies may hold potential for treating COVID-19-related OD, particularly as they have shown favorable results in some objective measures of olfactory function. However, given that evidence for some interventions remains limited and that some findings are influenced by study design and indirect comparisons, the conclusions should be interpreted with caution and remain to be further validated through additional high-quality RCTs.

## Informed consent and patient details

This is a network meta-analysis based on data extracted from previously published studies. Therefore, individual informed consent was not required for this secondary analysis. All primary studies included in this review had obtained informed consent from their participants.

## The ethics committee information and study protocol number

As this study is a secondary analysis of published aggregate data, it did not involve direct patient contact or intervention. Therefore, ethical approval and protocol registration by an institutional review board were not required. The study protocol was registered with PROSPERO (CRD420251117459).

## Funding

The authors declare that they did not receive any funding from any source.

## Conflicts of interest

The authors declare no conflicts of interest.

## Data Availability

The datasets generated and/or analyzed during the current study are available from the corresponding author on reasonable request.

## Appendix Supplementary materials

Image, application 1
