# Supplementary material for: Impacts of different treatment methods on patients with COVID-related olfactory dysfunction: a systematic review and network meta-analysis
Source: Clinics (Sao Paulo). 2026 Sep 8;81:101103. doi: 10.1016/j.clinsp.2026.101103 (PMC13573714; doi:10.1016/j.clinsp.2026.101103)
Supplement: Supplementary file 1 [file mmc1.doc]

CLINICS-D-26-00096_Supplementary Material

**Table S1** Database search strategy.

| **Pubmed** | | |
| --- | --- | --- |
| **#** | **Query** | **Results** |
| #1 | COVID-19[MeSH Terms] | 297,714 |
| #2 | "severe acute respiratory syndrome"[Title/Abstract] OR "SARS-CoV-2 Infection"[Title/Abstract] OR "SARS-CoV-2 disease"[Title/Abstract] OR "SARS Coronavirus 2 Infection"[Title/Abstract] OR "paucisymptomatic coronavirus disease 2019"[Title/Abstract] OR "novel coronavirus infection 2019"[Title/Abstract] OR "novel coronavirus disease 2019"[Title/Abstract] OR "nCoV 2019 infection"[Title/Abstract] OR "nCoV 2019 disease"[Title/Abstract] OR "COVID19"[Title/Abstract] OR "coronavirus infection 2019"[Title/Abstract] OR "Coronavirus Disease"[Title/Abstract] OR "2019 novel coronavirus epidemic"[Title/Abstract] | 426,911 |
| #3 | Olfaction Disorders[MeSH Terms] | 6,722 |
| #4 | "smelling disorder"[Title/Abstract] OR "smelling deprivation"[Title/Abstract] OR "Smell Loss"[Title/Abstract] OR "Smell Dysfunction"[Title/Abstract] OR "Smell Disorder"[Title/Abstract] OR "Phantosmia"[Title/Abstract] OR "Parosmia"[Title/Abstract] OR "Paraosmia"[Title/Abstract] OR "Olfactory Impairment"[Title/Abstract] OR "olfactory dysfunction"[Title/Abstract] OR "olfactory disturbance"[Title/Abstract] OR "olfactory distortion"[Title/Abstract] OR "olfactory disease"[Title/Abstract] OR "olfactory deprivation"[Title/Abstract] OR "olfactory anomaly"[Title/Abstract] OR "olfaction dysfunction"[Title/Abstract] OR "olfaction disorder"[Title/Abstract] OR "occupational smell disorder"[Title/Abstract] OR "loss of smell"[Title/Abstract] OR "Impaired Olfaction"[Title/Abstract] OR "Hyposmia"[Title/Abstract] OR "Dysosmia"[Title/Abstract] OR "disorder of smell"[Title/Abstract] OR "disorder of olfaction"[Title/Abstract] OR "Cacosmia"[Title/Abstract] OR "anosmy"[Title/Abstract] OR "anosmia"[Title/Abstract] OR "anodmia"[Title/Abstract] | 10,666 |
| #5 | Olfactory Training[MeSH Terms] | 49 |
| #6 | "smell training"[Title/Abstract] OR "smell retraining"[Title/Abstract] OR "smell rehabilitation"[Title/Abstract] OR "Olfactory Training"[Title/Abstract] OR "olfactory retraining"[Title/Abstract] OR "Olfactory Rehabilitation"[Title/Abstract] OR "Platelet-Rich Plasma"[Title/Abstract] OR "Palmitoylethanolamide"[Title/Abstract] OR "Luteolin"[Title/Abstract] OR "Vitamin A"[Title/Abstract] OR "Sodium Citrate"[Title/Abstract] OR "Omega-3"[Title/Abstract] OR "Nerve Growth Factor"[Title/Abstract] OR "Nasal Theophylline"[Title/Abstract] OR "Nasal Spray"[Title/Abstract] OR "Nasal Irrigation"[Title/Abstract] OR "Laser Treatment"[Title/Abstract] OR "Intranasal Photobiomodulation"[Title/Abstract] OR "Intranasal Insulin Films"[Title/Abstract] OR "Gabapentin"[Title/Abstract] OR "Forskolin"[Title/Abstract] OR "Corticosteroid"[Title/Abstract] OR "chelating agent"[Title/Abstract] OR "B-complex vitamins"[Title/Abstract] OR "Aromastick"[Title/Abstract] OR "Anodal transcranial Direct Current Stimulation"[Title/Abstract] OR "Alpha-lipoic acid"[Title/Abstract] OR "Acupuncture"[Title/Abstract] OR "Acupressure"[Title/Abstract] | 248,700 |
| #7 | ((#1 OR #2) AND (#3 OR #4)) AND (#5 OR #6) | 241 |
| **Embase** | | |
| **#** | **Query** | **Results** |
| #1 | 'coronavirus disease 2019'/exp | 477643 |
| #2 | 'severe acute respiratory syndrome':ti,ab,kw OR 'sars-cov-2 infection':ti,ab,kw OR 'sars-cov-2 disease':ti,ab,kw OR 'sars coronavirus 2 infection':ti,ab,kw OR 'paucisymptomatic coronavirus disease 2019':ti,ab,kw OR 'novel coronavirus infection 2019':ti,ab,kw OR 'novel coronavirus disease 2019':ti,ab,kw OR 'ncov 2019 infection':ti,ab,kw OR 'ncov 2019 disease':ti,ab,kw OR 'covid19':ti,ab,kw OR 'coronavirus infection 2019':ti,ab,kw OR 'coronavirus disease':ti,ab,kw OR '2019 novel coronavirus epidemic':ti,ab,kw | 507964 |
| #3 | 'smelling disorder'/exp | 24820 |
| #4 | 'smelling disorder':ti,ab,kw OR 'smelling deprivation':ti,ab,kw OR 'smell loss':ti,ab,kw OR 'smell dysfunction':ti,ab,kw OR 'smell disorder':ti,ab,kw OR 'phantosmia':ti,ab,kw OR 'parosmia':ti,ab,kw OR 'paraosmia':ti,ab,kw OR 'olfactory impairment':ti,ab,kw OR 'olfactory dysfunction':ti,ab,kw OR 'olfactory disturbance':ti,ab,kw OR 'olfactory distortion':ti,ab,kw OR 'olfactory disease':ti,ab,kw OR 'olfactory deprivation':ti,ab,kw OR 'olfactory anomaly':ti,ab,kw OR 'olfaction dysfunction':ti,ab,kw OR 'olfaction disorder':ti,ab,kw OR 'occupational smell disorder':ti,ab,kw OR 'loss of smell':ti,ab,kw OR 'impaired olfaction':ti,ab,kw OR 'hyposmia':ti,ab,kw OR 'dysosmia':ti,ab,kw OR 'disorder of smell':ti,ab,kw OR 'disorder of olfaction':ti,ab,kw OR 'cacosmia':ti,ab,kw OR 'anosmy':ti,ab,kw OR 'anosmia':ti,ab,kw OR 'anodmia':ti,ab,kw | 15388 |
| #5 | 'olfactory training'/exp | 304 |
| #6 | 'smell training':ti,ab,kw OR 'smell retraining':ti,ab,kw OR 'smell rehabilitation':ti,ab,kw OR 'olfactory training':ti,ab,kw OR 'olfactory retraining':ti,ab,kw OR 'olfactory rehabilitation':ti,ab,kw OR 'platelet-rich plasma':ti,ab,kw OR 'palmitoylethanolamide':ti,ab,kw OR 'luteolin':ti,ab,kw OR 'vitamin a':ti,ab,kw OR 'sodium citrate':ti,ab,kw OR 'omega-3':ti,ab,kw OR 'nerve growth factor':ti,ab,kw OR 'nasal theophylline':ti,ab,kw OR 'nasal spray':ti,ab,kw OR 'nasal irrigation':ti,ab,kw OR 'laser treatment':ti,ab,kw OR 'intranasal photobiomodulation':ti,ab,kw OR 'intranasal insulin films':ti,ab,kw OR 'gabapentin':ti,ab,kw OR 'forskolin':ti,ab,kw OR 'corticosteroid':ti,ab,kw OR 'chelating agent':ti,ab,kw OR 'b-complex vitamins':ti,ab,kw OR 'aromastick':ti,ab,kw OR 'anodal transcranial direct current stimulation':ti,ab,kw OR 'alpha-lipoic acid':ti,ab,kw OR 'acupuncture':ti,ab,kw OR 'acupressure':ti,ab,kw | 344116 |
| #7 | ((#1 OR #2) AND (#3 OR #4)) AND (#5 OR #6) | 392 |
| **Cochrane Library** | | |
| **#** | **Query** | **Results** |
| #1 | MeSH descriptor: [COVID-19] explode all trees | 8716 |
| #2 | ('severe acute respiratory syndrome' OR 'SARS-CoV-2 Infection' OR 'SARS-CoV-2 disease' OR 'SARS Coronavirus 2 Infection' OR 'paucisymptomatic coronavirus disease 2019' OR 'novel coronavirus infection 2019' OR 'novel coronavirus disease 2019' OR 'nCoV 2019 infection' OR 'nCoV 2019 disease' OR 'COVID19' OR 'coronavirus infection 2019' OR 'Coronavirus Disease' OR '2019 novel coronavirus epidemic'):ti,ab,kw | 13848 |
| #3 | MeSH descriptor: [Olfaction Disorders] explode all trees | 282 |
| #4 | ('smelling disorder' OR 'smelling deprivation' OR 'Smell Loss' OR 'Smell Dysfunction' OR 'Smell Disorder' OR 'Phantosmia' OR 'Parosmia' OR 'Paraosmia' OR 'Olfactory Impairment' OR 'olfactory dysfunction' OR 'olfactory disturbance' OR 'olfactory distortion' OR 'olfactory disease' OR 'olfactory deprivation' OR 'olfactory anomaly' OR 'olfaction dysfunction' OR 'olfaction disorder' OR 'occupational smell disorder' OR 'loss of smell' OR 'Impaired Olfaction' OR 'Hyposmia' OR 'Dysosmia' OR 'disorder of smell' OR 'disorder of olfaction' OR 'Cacosmia' OR 'anosmy' OR 'anosmia' OR 'anodmia'):ti,ab,kw | 1834 |
| #5 | MeSH descriptor: [Olfactory Training] explode all trees | 15 |
| #6 | ('smell training' OR 'smell retraining' OR 'smell rehabilitation' OR 'Olfactory Training' OR 'olfactory retraining' OR 'Olfactory Rehabilitation' OR 'Platelet-Rich Plasma' OR 'Palmitoylethanolamide' OR 'Luteolin' OR 'Vitamin A' OR 'Sodium Citrate' OR 'Omega-3' OR 'Nerve Growth Factor' OR 'Nasal Theophylline' OR 'Nasal Spray' OR 'Nasal Irrigation' OR 'Laser Treatment' OR 'Intranasal Photobiomodulation' OR 'Intranasal Insulin Films' OR 'Gabapentin' OR 'Forskolin' OR 'Corticosteroid' OR 'chelating agent' OR 'B-complex vitamins' OR 'Aromastick' OR 'Anodal transcranial Direct Current Stimulation' OR 'Alpha-lipoic acid' OR 'Acupuncture' OR 'Acupressure'):ti,ab,kw | 109973 |
| #7 | ((#1 OR #2) AND (#3 OR #4)) AND (#5 OR #6) | 141 |
| **Web of Science** | | |
| **#** | **Query** | **Results** |
| #1 | TS=("severe acute respiratory syndrome" OR "SARS-CoV-2 Infection" OR "SARS-CoV-2 disease" OR "SARS Coronavirus 2 Infection" OR "paucisymptomatic coronavirus disease 2019" OR "novel coronavirus infection 2019" OR "novel coronavirus disease 2019" OR "nCoV 2019 infection" OR "nCoV 2019 disease" OR "COVID19" OR "coronavirus infection 2019" OR "Coronavirus Disease" OR "2019 novel coronavirus epidemic") | 148250 |
| #2 | TS=("smelling disorder" OR "smelling deprivation" OR "Smell Loss" OR "Smell Dysfunction" OR "Smell Disorder" OR "Phantosmia" OR "Parosmia" OR "Paraosmia" OR "Olfactory Impairment" OR "olfactory dysfunction" OR "olfactory disturbance" OR "olfactory distortion" OR "olfactory disease" OR "olfactory deprivation" OR "olfactory anomaly" OR "olfaction dysfunction" OR "olfaction disorder" OR "occupational smell disorder" OR "loss of smell" OR "Impaired Olfaction" OR "Hyposmia" OR "Dysosmia" OR "disorder of smell" OR "disorder of olfaction" OR "Cacosmia" OR "anosmy" OR "anosmia" OR "anodmia”) | 9703 |
| #3 | TS=("smell training" OR "smell retraining" OR "smell rehabilitation" OR "Olfactory Training" OR "olfactory retraining" OR "Olfactory Rehabilitation" OR "Platelet-Rich Plasma" OR "Palmitoylethanolamide" OR "Luteolin" OR "Vitamin A" OR "Sodium Citrate" OR "Omega-3" OR "Nerve Growth Factor" OR "Nasal Theophylline" OR "Nasal Spray" OR "Nasal Irrigation" OR "Laser Treatment" OR "Intranasal Photobiomodulation" OR "Intranasal Insulin Films" OR "Gabapentin" OR "Forskolin" OR "Corticosteroid" OR "chelating agent" OR "B-complex vitamins" OR "Aromastick" OR "Anodal transcranial Direct Current Stimulation" OR "Alpha-lipoic acid" OR "Acupuncture" OR "Acupressure") | 207243 |
| #4 | #1 AND #2 | 1793 |
| #5 | #4 AND #3 | 95 |

**Table S2** Quality assessment.

(A)NIH

| **Figueiredo 2024** | Y | Y | NR | Y | N R | Y | N | Y | Y | Y | Y | Y | Y | NR | Fair |
| --- | --- | --- | --- | --- | --- | --- | --- | --- | --- | --- | --- | --- | --- | --- | --- |
| **Filiz 2024** | Y | NR | NR | NR | NR | Y | Y | Y | Y | Y | Y | NR | Y | NR | Fair |
| **Masson 2025** | Y | NR | NR | N | NR | Y | N | N | NR | Y | Y | Y | Y | N | Fair |
| **Tragoonrungsea 2023** | Y | Y | Y | N | Y | Y | Y | Y | NR | Y | Y | Y | Y | NR | Good |
| **Abdelalim 2021** | Y | NR | NR | N | NR | Y | Y | Y | NR | Y | Y | NR | Y | N | Fair |
| **Lechner 2022** | Y | NR | NR | N | NR | Y | Y | Y | NR | Y | Y | Y | Y | N | Fair |
| **Abdelazim a 2022** | Y | Y | Y | Y | Y | Y | Y | Y | NR | Y | Y | NR | Y | Y | Good |
| **Abdelazim b 2022** | Y | Y | Y | Y | NR | Y | Y | Y | NR | Y | Y | NR | Y | Y | Good |
| **Mogensen 2025** | Y | Y | Y | N | NR | Y | N | Y | Y | Y | Y | Y | Y | N | Fair |
| **Abdelazim c 2022** | Y | Y | Y | Y | NR | Y | Y | Y | NR | Y | Y | NR | NR | Y | Fair |
| **Steffens 2022** | N | N | N | N | NR | Y | Y | N | NR | Y | Y | NR | NR | NR | Poor |
| **Mohebbi 2024** | Y | Y | NR | N | NR | Y | Y | Y | Y | Y | Y | NR | Y | Y | Fair |
| **Khan 2023** | Y | Y | NR | N | NR | Y | N | Y | Y | Y | Y | Y | Y | Y | Fair |
| **Abdelazim 2024** | Y | Y | NR | Y | NR | Y | Y | Y | NR | Y | Y | NR | Y | N | Fair |
| **Mahadev 2023** | Y | Y | NR | Y | NR | Y | N | N | Y | Y | Y | NR | Y | N | Fair |
| **Imam 2023** | Y | Y | Y | Y | NR | Y | Y | Y | NR | Y | Y | NR | Y | Y | Good |
| **Altemani 2024** | Y | Y | Y | Y | NR | Y | Y | Y | NR | Y | Y | Y | Y | Y | Good |
| **Oliveira 2025** | Y | Y | Y | N | Y | N | Y | Y | NR | Y | Y | Y | Y | N | Fair |
| **Armstrong 2025** | Y | NR | NR | N | Y | Y | N | N | NR | Y | Y | NR | NR | N | Fair |
| **Pires 2022** | Y | Y | NR | N | NR | Y | Y | Y | Y | Y | Y | NR | Y | Y | Fair |
| **Abdelazim 2023** | Y | Y | NR | N | Y | Y | Y | Y | Y | Y | Y | NR | Y | Y | Good |
| **Imam 2025** | Y | Y | Y | Y | Y | Y | Y | Y | NR | Y | Y | NR | Y | Y | Good |
| **Hosseinpoor 2022** | Y | Y | Y | Y | NR | Y | Y | Y | NR | Y | Y | Y | Y | N | Good |
| **Kasiri 2021** | Y | Y | NR | Y | Y | Y | Y | Y | NR | Y | Y | NR | Y | NR | Fair |
| **Berube 2023** | Y | Y | Y | Y | Y | Y | Y | Y | NR | Y | Y | Y | Y | N | Good |
| **Lerner 2022** | Y | Y | NR | Y | Y | Y | Y | Y | NR | Y | Y | Y | Y | N | Good |
| **Hernandez 2022** | Y | NR | NR | N | NR | Y | Y | Y | Y | Y | Y | NR | Y | NR | Fair |
| **Gellrich 2024** | Y | NR | NR | N | NR | N | N | N | NR | Y | Y | NR | Y | NR | Poor |
| **Stadio 2023** | Y | NR | NR | Y | Y | N | Y | Y | NR | Y | Y | NR | Y | Y | Fair |
| **Cantone 2024** | Y | Y | NR | Y | Y | N | N | N | NR | Y | Y | NR | Y | NR | Fair |
| **Chung 2023** | Y | Y | N | N | Y | Y | Y | Y | NR | Y | Y | Y | Y | NR | Fair |
| **Schepens 2022** | Y | Y | Y | Y | NR | Y | Y | Y | Y | Y | Y | Y | Y | Y | Good |
| **Vestito 2025** | Y | Y | NR | Y | Y | Y | Y | Y | NR | Y | Y | Y | Y | NR | Good |
| **Mohamad 2022** | Y | Y | Y | Y | Y | Y | Y | Y | NR | Y | Y | NR | Y | NR | Good |
| **Algarni 2025** | Y | Y | Y | Y | Y | Y | Y | Y | NR | Y | Y | Y | Y | Y | Good |
| **Duffy 2024** | Y | Y | NR | N | NR | Y | Y | Y | NR | Y | Y | Y | Y | Y | Fair |

(B) NOS

| **Study** | | **DeLuca 2022** | **Pendolino 2025** | **Lechien 2024** | **Yaylacı 2023** | **Le Bon 2021** | **Fieux 2025** | **Pendolino 2023** | **Genetzaki 2021** |
| --- | --- | --- | --- | --- | --- | --- | --- | --- | --- |
| Selection | Representativeness of the exposed cohort | **1** | **1** | **1** | **1** | **1** | **1** | **1** | **1** |
|  | Selection of the nonexposed cohort | **1** | **1** | **0** | **1** | **0** | **1** | **1** | **1** |
|  | Ascertainment of exposure | **1** | **1** | **1** | **1** | **1** | **1** | **1** | **1** |
|  | Demonstration that outcome of interest wae not present at start og study | **1** | **1** | **1** | **1** | **1** | **1** | **1** | **1** |
| Comparability | Comparability of cohorts on the basis of the design or analysis | **1** | **1** | **1** | **1** | **0** | **2** | **1** | **0** |
| Outcome | Assessment of outcome | **1** | **1** | **1** | **1** | **1** | **1** | **1** | **1** |
|  | Was follow up long enough for outcomes to occur | **1** | **1** | **1** | **1** | **1** | **1** | **1** | **1** |
|  | Adequacy of follow up of cohorts | **0** | **1** | **0** | **1** | **1** | **1** | **0** | **1** |
| Quality scores |  | **Good** | **Good** | **Fair** | **Good** | **Fair** | **Good** | **Good** | **Good** |

**Table** **S3** League table.

(a) League table of T of different treatment methods in network meta-analysis.

| **Chelating agent** |  |  |  |  |  |  |  |  |  |
| --- | --- | --- | --- | --- | --- | --- | --- | --- | --- |
| 1.15 (-1.88, 4.28) | **OT + Chelating agent** |  |  |  |  |  |  |  |  |
| 1.73 (-2.15, 5.77) | 0.6 (-3.93, 5.15) | **FSPR** |  |  |  |  |  |  |  |
| 2.08 (-2.12, 6.44) | 0.94 (-3.55, 5.43) | 0.35 (-5.06, 5.76) | **OT + Omega-3** |  |  |  |  |  |  |
| 2.09 (-1.69, 6.08) | 0.95 (-3.49, 5.48) | 0.35 (-4.73, 5.51) | 0 (-5.36, 5.41) | **PRP** |  |  |  |  |  |
| 2.36 (-1.84, 6.7) | 1.22 (-3.29, 5.69) | 0.62 (-4.79, 6.02) | 0.27 (-4.83, 5.39) | 0.26 (-5.18, 5.62) | **OT + PEA-LUT** |  |  |  |  |
| **2.55 (0.32, 4.93)** | 1.4 (-1.27, 4.1) | 0.8 (-3.24, 4.88) | 0.46 (-3.13, 4.03) | 0.46 (-3.58, 4.43) | 0.2 (-3.41, 3.82) | **OT** |  |  |  |
| 2.71 (-1.08, 6.62) | 1.56 (-2.92, 6.01) | 0.97 (-4.12, 5.98) | 0.62 (-4.78, 5.95) | 0.61 (-4.47, 5.57) | 0.35 (-5, 5.69) | 0.16 (-3.85, 4.08) | **Forskolin** |  |  |
| **2.88 (0.14, 5.76)** | 1.74 (-1.56, 5.04) | 1.14 (-3.2, 5.53) | 0.79 (-3.41, 4.99) | 0.78 (-3.54, 5.08) | 0.52 (-3.67, 4.73) | 0.33 (-1.85, 2.49) | 0.17 (-4.08, 4.48) | **OT + Corticosteroid** |  |
| **2.89 (1.47, 4.45)** | 1.74 (-0.94, 4.45) | 1.15 (-2.5, 4.81) | 0.8 (-3.2, 4.82) | 0.8 (-2.82, 4.33) | 0.53 (-3.49, 4.58) | 0.34 (-1.44, 2.11) | 0.18 (-3.35, 3.78) | 0.01 (-2.38, 2.41) | **Control** |

Note: OT, Olfactory Training; FSPR, Functional Septorhinoplasty; PRP, platelet-rich plasma; PEA-LUT, Palmitoylethanolamide and Luteolin.

**(b) League table of D of different treatment methods in network meta-analysis.**

| **Chelating agent** |  |  |  |  |  |  |  |  |  |  |  |  |
| --- | --- | --- | --- | --- | --- | --- | --- | --- | --- | --- | --- | --- |
| 0.43 (-0.6, 1.51) | **Corticosteroid + Antihistamine** |  |  |  |  |  |  |  |  |  |  |  |
| 0.48 (-0.61, 1.6) | 0.05 (-1.26, 1.35) | **OT + Chelating agent** |  |  |  |  |  |  |  |  |  |  |
| 0.94 (-0.09, 2.01) | 0.51 (-0.39, 1.4) | 0.45 (-0.85, 1.77) | **Corticosteroid** |  |  |  |  |  |  |  |  |  |
| 1.17 (-0.16, 2.56) | 0.74 (-0.79, 2.27) | 0.69 (-0.87, 2.27) | 0.23 (-1.28, 1.77) | **Forskolin** |  |  |  |  |  |  |  |  |
| 1.43 (-0.17, 3.08) | 0.99 (-0.75, 2.76) | 0.94 (-0.83, 2.76) | 0.49 (-1.27, 2.27) | 0.25 (-1.68, 2.22) | **OT + PEA-LUT** |  |  |  |  |  |  |  |
| **1.47 (0.12, 2.86)** | 1.03 (-0.5, 2.58) | 0.98 (-0.59, 2.57) | 0.53 (-1.01, 2.07) | 0.29 (-1.46, 2.05) | 0.04 (-1.95, 2.01) | **PRP** |  |  |  |  |  |  |
| **1.59 (0.56, 2.65)** | **1.16 (0.25, 2.05)** | 1.11 (-0.21, 2.42) | 0.65 (-0.25, 1.55) | 0.42 (-1.11, 1.94) | 0.16 (-1.61, 1.91) | 0.12 (-1.43, 1.65) | **Antihistamine** |  |  |  |  |  |
| **1.66 (0.09, 3.28)** | 1.23 (-0.51, 3) | 1.18 (-0.6, 2.97) | 0.72 (-1.02, 2.47) | 0.48 (-1.46, 2.42) | 0.23 (-1.66, 2.1) | 0.19 (-1.76, 2.16) | 0.07 (-1.67, 1.82) | **OT + Omega-3** |  |  |  |  |
| **1.65 (0.58, 2.75)** | 1.22 (-0.08, 2.51) | 1.17 (-0.17, 2.53) | 0.71 (-0.57, 2.01) | 0.47 (-1.06, 2.02) | 0.22 (-1.37, 1.78) | 0.18 (-1.37, 1.75) | 0.06 (-1.23, 1.37) | -0.01 (-1.56, 1.53) | **OT + Corticosteroid** |  |  |  |
| **1.79 (0.9, 2.72)** | **1.36 (0.21, 2.5)** | **1.3 (0.1, 2.52)** | 0.85 (-0.29, 2) | 0.61 (-0.81, 2.04) | 0.36 (-0.99, 1.7) | 0.32 (-1.12, 1.78) | 0.2 (-0.94, 1.36) | 0.13 (-1.18, 1.44) | 0.14 (-0.67, 0.95) | **OT** |  |  |
| **1.92 (0.4, 3.47)** | 1.49 (-0.21, 3.17) | 1.43 (-0.29, 3.16) | 0.98 (-0.72, 2.68) | 0.75 (-1.13, 2.62) | 0.49 (-1.6, 2.58) | 0.45 (-1.46, 2.35) | 0.33 (-1.36, 2.01) | 0.26 (-1.81, 2.31) | 0.27 (-1.44, 1.97) | 0.13 (-1.48, 1.72) | **FSPR** |  |
| **1.93 (1.38, 2.5)** | **1.49 (0.6, 2.39)** | **1.44 (0.49, 2.41)** | **0.9 9(0.09, 1.88)** | 0.75 (-0.49, 1.98) | 0.5 (-1.03, 2.01) | 0.46 (-0.79, 1.71) | 0.33 (-0.55, 1.23) | 0.26 (-1.24, 1.76) | 0.27 (-0.66, 1.21) | 0.14 (-0.59, 0.85) | 0.01 (-1.43, 1.44) | **Control** |

Note: OT, Olfactory Training; PEA-LUT, Palmitoylethanolamide and Luteolin; PRP, Platelet-Rich Plasma; FSPR, Functional Septorhinoplasty.

**(c) League table of I of different treatment methods in network meta-analysis.**

| **Chelating agent** |  |  |  |  |  |  |  |  |  |
| --- | --- | --- | --- | --- | --- | --- | --- | --- | --- |
| 1.09 (-0.22, 2.4) | **OT + Chelating agent** |  |  |  |  |  |  |  |  |
| **1.53 (0.12, 3)** | 0.43 (-1.08, 2.02) | **OT + PEA-LUT** |  |  |  |  |  |  |  |
| **2.16 (1.2, 3.17)** | 1.07 (-0.04, 2.24) | 0.63 (-0.43, 1.68) | **OT** |  |  |  |  |  |  |
| **2.17 (0.62, 3.74)** | 1.07 (-0.71, 2.94) | 0.64 (-1.3, 2.55) | 0.01 (-1.61, 1.61) | **Forskolin** |  |  |  |  |  |
| **2.21 (0.51, 3.95)** | 1.12 (-0.82, 3.11) | 0.69 (-1.38, 2.72) | 0.05 (-1.72, 1.81) | 0.04 (-2.08, 2.19) | **FSPR** |  |  |  |  |
| **2.31 (1.11, 3.52)** | 1.21 (-0.18, 2.63) | 0.78 (-0.64, 2.14) | 0.14 (-0.78, 1.04) | 0.14 (-1.62, 1.88) | 0.09 (-1.81, 2) | **OT + Corticosteroid** |  |  |  |
| **2.45 (0.89, 4.03)** | 1.36 (-0.46, 3.2) | 0.92 (-1.01, 2.83) | 0.29 (-1.33, 1.9) | 0.28 (-1.74, 2.3) | 0.24 (-1.93, 2.38) | 0.15 (-1.61,1.9) | **PRP** |  |  |
| **2.56 (0.79, 4.37)** | 1.47 (-0.4, 3.37) | 1.03 (-0.82, 2.86) | 0.4 (-1.1, 1.88) | 0.39 (-1.79, 2.59) | 0.35 (-1.97, 2.66) | 0.26 (-1.48, 2.01) | 0.11 (-2.08, 2.32) | **OT + Omega-3** |  |
| **2.64 (2.02, 3.29)** | **1.54 (0.41, 2.72)** | 1.11 (-0.2, 2.39) | 0.48 (-0.29, 1.22) | 0.47 (-0.96, 1.89) | 0.43 (-1.18, 2.03) | 0.34 (-0.69, 1.36) | 0.19 (-1.24, 1.62) | 0.08 (-1.59, 1.75) | **Control** |

Note: OT, Olfactory Training; PEA-LUT, Palmitoylethanolamide and Luteolin; FSPR, Functional Septorhinoplasty; PRP, Platelet-Rich Plasma.

**(d) League table of VAS of different treatment methods in network meta-analysis.**

| **Corticosteroid** |  |  |  |  |  |  |  |  |  |  |  |
| --- | --- | --- | --- | --- | --- | --- | --- | --- | --- | --- | --- |
| 0.64 (-1.98, 3.31) | **PRP** |  |  |  |  |  |  |  |  |  |  |
| 0.78 (-1.88, 3.48) | 0.14 (-2.94, 3.28) | **OT + Infrared PBMT + Corticosteroid** |  |  |  |  |  |  |  |  |  |
| 1.36 (-1.24, 3.97) | 0.72 (-2.37, 3.75) | 0.57 (-2.53, 3.65) | **TCM** |  |  |  |  |  |  |  |  |
| 1.39 (-1.29, 4.09) | 0.75 (-2.37, 3.88) | 0.61 (-1.12, 2.31) | 0.03 (-3.05, 3.15) | **OT + Red-light + Corticosteroid** |  |  |  |  |  |  |  |
| 1.51 (-1.53, 4.56) | 0.87 (-2.57, 4.26) | 0.72 (-2.09, 3.54) | 0.15 (-3.27, 3.56) | 0.12 (-2.68 ,2.91) | **OT + Corticosteroid + TCM** |  |  |  |  |  |  |
| 1.57 (-0.87, 4.03) | 0.92 (-1.99, 3.86) | 0.78 (-1.69, 3.25) | 0.2 (-2.68, 3.12) | 0.17 (-2.27, 2.65) | 0.06 (-2.77, 2.9) | **Strengthening OT** |  |  |  |  |  |
| 1.6 (-1.04, 4.22) | 0.96 (-2.12, 4.02) | 0.82 (-2.33, 3.91) | 0.24 (-2.81, 3.3) | 0.21 (-2.93, 3.32) | 0.08 (-3.3, 3.49) | 0.04 (-2.93, 2.92) | **FSPR** |  |  |  |  |
| 1.59 (-0.43, 3.66) | 0.95 (-1.65, 3.56) | 0.81 (-0.91, 2.53) | 0.23 (-2.31, 2.83) | 0.2 (-1.49, 1.92) | 0.09 (-2.16, 2.32) | 0.03 (-1.75, 1.79) | -0.01 (-2.57, 2.62) | **OT + Corticosteroid** |  |  |  |
| 1.77 (-1.07, 4.62) | 1.12 (-2.16, 4.36) | 0.98 (-1.89, 3.84) | 0.4 (-2.82, 3.67) | 0.37 (-2.49, 3.25) | 0.27 (-2.97, 3.48) | 0.2 (-2.36, 2.75) | 0.17 (-3.07, 3.45) | 0.17 (-2.15, 2.45) | **OT + ALA** |  |  |
| 1.77 (-0.18, 3.72) | 1.13 (-1.41, 3.64) | 0.98 (-1.01, 2.96) | 0.4 (-2.07, 2.94) | 0.37 (-1.59, 2.35) | 0.26 (-2.19, 2.7) | 0.2 (-1.29, 1.68) | 0.16 (-2.34, 2.72) | 0.17 (-0.81, 1.15) | 0 (-2.08, 2.09) | **OT** |  |
| **1.99 (0.52, 3.48)** | 1.35 (-0.85, 3.51) | 1.21 (-1.03, 3.43) | 0.63 (-1.49, 2.79) | 0.6 (-1.64, 2.81) | 0.48 (-2.15, 3.14) | 0.43 (-1.53, 2.38) | 0.39 (-1.77, 2.57) | 0.39 (-1.02, 1.82) | 0.23 (-2.2, 2.69) | 0.23 (-1.05, 1.51) | **Control** |

Note: OT, Olfactory Training; PRP, Platelet-Rich Plasma; TCM, Traditional Chinese Medicine external treatment; PEA-LUT, Palmitoylethanolamide and Luteolin; FSPR, Functional Septorhinoplasty; ALA, Alpha-Lipoic Acid.

**Table S4** Type of chelating agents included in the network meta-analysis.

| **Study** | **Specific chelating agent** | **Mean ∆TDI ± SD (vs. baseline)** | **Mean ∆ nasal calcium concentration ± SD** | **Main pharmacologic feature** |
| --- | --- | --- | --- | --- |
| Abdelazim a 2022 | Nitrilotriacetic acid trisodium salt | 20.33 ±1.42 | -25.75±2.43 | Chelates free calcium ions in nasal mucus; may reduce calcium-mediated inhibition of olfactory signal transduction. |
| Abdelazim b 2022 | Tetra sodium pyrophosphate | 5.41 ± 2.52 | -8.44 ± 4.71 | Calcium-chelating and buffering agent; may reduce local calcium concentration in nasal secretions. |
| Abdelazim c 2022 | Sodium gluconate | 5.24 ± 2.35 | -12.00 ± 5.40 | Chelating agent that forms stable complexes with metal ions; may reduce calcium-related interference in olfactory function. |
| Imam 2023 | Pentasodium diethylenetriamine pentaacetate | 8.64 ±2.84 | -13.15±3.92 | Chelates divalent and trivalent metal ions, including calcium; may modulate the local ionic environment. |
| Altemani 2024 | Sodium phytate | 5.76 ± 3.18 | -12.43 ± 7.10 | Natural calcium-binding agent; may regulate the local calcium environment and reduce calcium-mediated inhibition. |
| Abdelazim 2023 | Ethylene diamine tetra acetic acid | 7.52 ±2.89 | -14.08±5.06 | Chelates divalent and trivalent cations such as calcium and magnesium; may reduce free calcium in nasal secretions. |
| Imam 2025 | Disodium orthophosphate | 10.84 ± 5.46 | -15.07 ± 4.89 | Calcium-chelating agent; may bind free calcium ions in nasal mucus and reduce inhibitory effects on olfactory signaling. |
| Algarni 2025 | Glutamate diacetate | / | -7.36±2.67 | Natural chelating agent with calcium-binding capacity; may lower local calcium levels. |

Note: TDI, Sniffin' Sticks test; SD, Standard Deviation; ∆, Change from baseline; /, Not mentioned.

**Table S5** League table that only includes RCTs.

(a) League table of TDI of different treatment methods in network meta-analysis.

| **Chelating agent** |  |  |  |  |  |  |  |  |  |  |
| --- | --- | --- | --- | --- | --- | --- | --- | --- | --- | --- |
| 1.03 (-3.01, 5.17) | OT + A-tDCS |  |  |  |  |  |  |  |  |  |
| 1.74 (-1.03, 4.62) | 0.71 (-3.17, 4.56) | OT + Chelating agent |  |  |  |  |  |  |  |  |
| 1.85 (-1.39, 5.18) | 0.82 (-2.66, 4.29) | 0.11 (-2.84, 3.08) | OT + PEA-LUT |  |  |  |  |  |  |  |
| 2.14 (-1.24, 5.63) | 1.11 (-2.53, 4.74) | 0.41 (-2.77, 3.57) | 0.3 (-1.75, 2.32) | OT + PEA-LUT + ALA |  |  |  |  |  |  |
| 2.82 (-0.35, 6.15) | 1.79 (-3.07, 6.7) | 1.09 (-2.73, 4.94) | 0.97 (-3.19, 5.17) | 0.68 (-3.66, 5) | Forskolin |  |  |  |  |  |
| 3.19 (-0.83, 7.3) | 2.16 (-2.09, 6.36) | 1.45 (-2.36, 5.33) | 1.34 (-2.07, 4.79) | 1.04 (-2.52, 4.64) | 0.36 (-4.47, 5.2) | OT + Corticosteroid |  |  |  |  |
| 2.99 (-1.02, 7.12) | 1.95 (-2.3, 6.18) | 1.26 (-2.6, 5.1) | 1.13 (-2.3, 4.58) | 0.84 (-2.73, 4.44) | 0.16 (-4.68, 5.01) | -0.21 (-4.4, 3.96) | OT + Omega-3 |  |  |  |
| 3.01 (-0.35, 6.51) | 1.98 (-1.65, 5.65) | 1.27 (-1.84, 4.43) | 1.16 (-0.87, 3.21) | 0.88 (-1.25, 3.01) | 0.19 (-4.09, 4.53) | -0.17 (-3.76, 3.41) | 0.03 (-3.58, 3.61) | OT+ALA |  |  |
| **3.07 (0.35, 5.93)** | 2.03 (-1, 5.04) | 1.33 (-1.09, 3.78) | 1.22 (-0.49, 2.95) | 0.92 (-1.1, 2.95) | 0.24 (-3.58, 4.1) | -0.12 (-3.08, 2.82) | 0.09 (-2.9, 3.06) | 0.05 (-1.97, 2.09) | OT |  |
| **3.72 (2.42, 5.16)** | 2.68 (-1.19, 6.56) | 1.98 (-0.47, 4.44) | 1.87 (-1.11, 4.87) | 1.57 (-1.58, 4.74) | 0.89 (-2.06, 3.82) | 0.52 (-3.3, 4.38) | 0.73 (-3.09, 4.6) | 0.7 (-2.46, 3.85) | 0.65 (-1.79, 3.07) | Control |

Note: OT, Olfactory Training; A-tDCS, Anodal Transcranial Direct Current stimulation; PEA-LUT, Palmitoylethanolamide and Luteolin; ALA, Alpha-Lipoic Acid.

**(b) League table of T of different treatment methods in network meta-analysis.**

| **Chelating agent** |  |  |  |  |  |  |  |
| --- | --- | --- | --- | --- | --- | --- | --- |
| 1.07 (-4.15, 6.37) | OT + Chelating agent |  |  |  |  |  |  |
| 1.89 (-5.85, 9.73) | 0.82 (-6.59, 8.17) | OT + Omega-3 |  |  |  |  |  |
| 2.16 (-5.58, 9.98) | 1.1 (-6.32, 8.5) | 0.27 (-7.79, 8.44) | OT + PEA-LUT |  |  |  |  |
| 2.36 (-2.84, 7.62) | 1.3 (-3.41, 5.97) | 0.48 (-5.23, 6.22) | 0.2 (-5.54, 5.92) | OT |  |  |  |
| 2.77 (-3.43, 9.04) | 1.7 (-5.73, 9.11) | 0.87 (-8.54, 10.31) | 0.62 (-8.71, 9.93) | 0.4 (-6.96, 7.75) | Forskolin |  |  |
| 2.31 (-5.45, 10.04) | 1.21 (-6.19, 8.72) | 0.41 (-7.7, 8.51) | 0.13 (-7.96, 8.3) | -0.06 (-5.86, 5.65) | -0.46 (-9.82, 8.81) | OT + Corticosteroid |  |
| **2.97 (0.66, 5.36)** | 1.89 (-2.79, 6.62) | 1.07 (-6.32, 8.51) | 0.81 (-6.59, 8.25) | 0.59 (-4.06, 5.33) | 0.2 (-5.54, 5.97) | 0.65 (-6.68, 8.08) | Control |

Note: OT, Olfactory Training; PEA-LUT, Palmitoylethanolamide and Luteolin.

**(c) League table of D of different treatment methods in network meta-analysis.**

| **Chelating agent** |  |  |  |  |  |  |  |  |  |  |
| --- | --- | --- | --- | --- | --- | --- | --- | --- | --- | --- |
| 0.44 (-0.8, 1.71) | Corticosteroid + Antihistamine |  |  |  |  |  |  |  |  |  |
| 0.49 (-0.79, 1.81) | 0.05 (-1.52, 1.59) | OT + Chelating agent |  |  |  |  |  |  |  |  |
| 0.95 (-0.28, 2.23) | 0.51 (-0.57, 1.59) | 0.46 (-1.08, 2.02) | Corticosteroid |  |  |  |  |  |  |  |
| 1.18 (-0.42, 2.83) | 0.74 (-1.09, 2.58) | 0.69 (-1.15, 2.54) | 0.23 (-1.6, 2.06) | Forskolin |  |  |  |  |  |  |
| 1.61 (-0.69, 3.94) | 1.17 (-1.3, 3.65) | 1.13 (-1.38, 3.63) | 0.66 (-1.81, 3.12) | 0.43 (-2.26, 3.11) | OT + PEA-LUT |  |  |  |  |  |
| **1.6 (0.36, 2.89)** | **1.16 (0.09, 2.24)** | 1.11 (-0.43, 2.66) | 0.65 (-0.42, 1.73) | 0.42 (-1.42, 2.25) | -0.01 (-2.48, 2.48) | Antihistamine |  |  |  |  |
| 1.84 (-0.41, 4.17) | 1.39 (-1.02, 3.88) | 1.35 (-1.09, 3.86) | 0.89 (-1.54, 3.37) | 0.66 (-1.98, 3.33) | 0.23 (-2.01, 2.49) | 0.24 (-2.19, 2.71) | OT + Omega-3 |  |  |  |
| 1.89 (-0.33, 4.19) | 1.45 (-0.95, 3.9) | 1.41 (-1.03, 3.87) | 0.94 (-1.46, 3.38) | 0.71 (-1.91, 3.37) | 0.28 (-1.91, 2.48) | 0.3 (-2.11, 2.75) | 0.06 (-2.13, 2.22) | OT + Corticosteroid |  |  |
| **1.97 (0.32, 3.69)** | 1.53 (-0.35, 3.43) | 1.48 (-0.44, 3.4) | 1.01 (-0.86, 2.91) | 0.79 (-1.38, 2.96) | 0.35 (-1.24, 1.95) | 0.37 (-1.52, 2.27) | 0.13 (-1.44, 1.69) | 0.07 (-1.44, 1.59) | OT |  |
| **1.93 (1.3, 2.6)** | **1.49 (0.42, 2.56)** | **1.44 (0.32, 2.57)** | 0.98 (-0.09, 2.05) | 0.75 (-0.73, 2.24) | 0.32 (-1.9, 2.56) | 0.34 (-0.75, 1.41) | 0.1 (-2.13, 2.28) | 0.04 (-2.14, 2.18) | -0.03 (-1.6, 1.51) | Control |

Note: OT, Olfactory Training; PEA-LUT, Palmitoylethanolamide and Luteolin.

**(d) League table of I of different treatment methods in network meta-analysis.**

| Chelating agent |  |  |  |  |  |  |  |
| --- | --- | --- | --- | --- | --- | --- | --- |
| 0.83 (-0.93, 2.58) | OT + Chelating agent |  |  |  |  |  |  |
| 1.06 (-1.09, 3.3) | 0.24 (-1.77, 2.32) | OT+PEA-LUT |  |  |  |  |  |
| 1.68 (-0.05, 3.44) | 0.85 (-0.69, 2.41) | 0.62 (-0.72, 1.93) | OT |  |  |  |  |
| **2.18 (0.22, 4.19)** | 1.35 (-1.02, 3.78) | 1.12 (-1.64, 3.82) | 0.5 (-1.88, 2.88) | Forskolin |  |  |  |
| 2.16 (-0.37, 4.7) | 1.32 (-1.07, 3.75) | 1.09 (-1.21, 3.34) | 0.47 (-1.39, 2.33) | -0.03 (-3.02, 2.99) | OT+Corticosteroid |  |  |
| 2.09 (-0.47, 4.69) | 1.26 (-1.17, 3.7) | 1.02 (-1.3, 3.3) | 0.4 (-1.47, 2.27) | -0.1 (-3.13, 2.95) | -0.06 (-2.72, 2.58) | OT+Omega-3 |  |
| **2.64 (1.87, 3.46)** | **1.81 (0.26, 3.41)** | 1.57 (-0.48, 3.61) | 0.96 (-0.58, 2.51) | 0.46 (-1.37, 2.28) | 0.49 (-1.92, 2.91) | 0.56 (-1.89, 2.99) | Control |

Note: OT, Olfactory Training; PEA-LUT, Palmitoylethanolamide and Luteolin.

**(e) League table of UPSIT of different treatment methods in network meta-analysis.**

| **PRP** |  |  |  |  |  |  |  |
| --- | --- | --- | --- | --- | --- | --- | --- |
| 0.47 (-0.43, 1.37) | OT+VitA |  |  |  |  |  |  |
| **1.12 (0.56, 1.67)** | 0.65 (-0.13, 1.44) | MOT |  |  |  |  |  |
| **1.15 (0.43, 1.86)** | 0.68 (-0.21, 1.57) | 0.03 (-0.49, 0.56) | Strengthening OT |  |  |  |  |
| **1.16 (0.55, 1.77)** | 0.7 (-0.15, 1.54) | 0.05 (-0.4, 0.5) | 0.02 (-0.61, 0.65) | Omega-3 |  |  |  |
| **1.25 (0.71, 1.78)** | **0.78 (0.02, 1.54)** | 0.13 (-0.11, 0.38) | 0.1 (-0.36, 0.57) | 0.08 (-0.34, 0.51) | OT |  |  |
| **1.43 (0.74, 2.11)** | **0.96 (0.06, 1.85)** | 0.31 (-0.23, 0.85) | 0.28 (-0.42, 0.98) | 0.26 (-0.33, 0.86) | 0.18 (-0.34, 0.7) | Gabapentin |  |
| **1.47 (0.98, 1.95)** | **1 (0.24, 1.76)** | **0.35 (0.09, 0.61)** | 0.32 (-0.19, 0.83) | 0.3 (-0.06, 0.67) | **0.22 (0, 0.43)** | 0.04 (-0.43, 0.51) | Control |

Note: PRP, Platelet-Rich Plasma; OT, Olfactory Training; VitA, Vitamin A; MOT, Multisensory Olfactory Training.

**(f) League table of VAS of different treatment methods in network meta-analysis.**

| **Corticosteroid** |  |  |  |  |  |  |  |  |  |
| --- | --- | --- | --- | --- | --- | --- | --- | --- | --- |
| 0.57 (-4.2, 5.32) | OT + Infrared PBMT + Corticosteroid |  |  |  |  |  |  |  |  |
| 1.37 (-2.34, 5.03) | 0.81 (-4.46, 6.03) | TCM |  |  |  |  |  |  |  |
| 1.16 (-3.6, 5.98) | 0.59 (-1.92, 3.07) | -0.21 (-5.42, 5.06) | OT + Red-light + Corticosteroid |  |  |  |  |  |  |
| 1.27 (-3.9, 6.48) | 0.71 (-3.29, 4.76) | -0.08 (-5.75, 5.53) | 0.11 (-3.86, 4.13) | OT + Corticosteroid + TCM |  |  |  |  |  |
| 1.36 (-2.94, 5.6) | 0.79 (-2.9, 4.47) | 0 (-4.83, 4.77) | 0.2 (-3.51, 3.87) | 0.07 (-4.1, 4.26) | Strengthening OT |  |  |  |  |
| 1.36 (-2.73, 5.46) | 0.8 (-1.69, 3.27) | 0 (-4.63, 4.64) | 0.21 (-2.25, 2.71) | 0.09 (-3.07, 3.21) | 0 (-2.73, 2.78) | OT + Corticosteroid |  |  |  |
| 1.57 (-3.2, 6.35) | 1.01 (-3.28, 5.23) | 0.19 (-5.02, 5.44) | 0.41 (-3.87, 4.64) | 0.29 (-4.42, 4.95) | 0.21 (-3.48, 3.87) | 0.21 (-3.29, 3.65) | OT+ALA |  |  |
| 1.57 (-2.15, 5.25) | 1 (-2, 4.01) | 0.19 (-4.13, 4.5) | 0.4 (-2.62, 3.39) | 0.29 (-3.32, 3.88) | 0.2 (-1.93, 2.34) | 0.2 (-1.55, 1.92) | -0.01 (-2.99, 3.03) | OT |  |
| 2 (-0.15, 4.13) | 1.43 (-2.85, 5.67) | 0.64 (-2.38, 3.66) | 0.84 (-3.46, 5.09) | 0.72 (-4.03, 5.42) | 0.64 (-3.05, 4.37) | 0.63 (-2.89, 4.12) | 0.45 (-3.87, 4.71) | 0.43 (-2.59, 3.47) | Control |

Note: OT, Olfactory Training; Infrared PBMT, Infrared Photobiomodulation Therapy; TCM, Taditional Chinese Medicine external treatment.

**Table S6** League table for total TDI after exclusion of each chelator-related study in the leave-one-out sensitivity analysis.

**(a) League table for total TDI after exclusion of Abdelazim a 2022 in the leave-one-out sensitivity analysis.**

| **Chelating agent** |  |  |  |  |  |  |  |  |  |  |  |  |  |
| --- | --- | --- | --- | --- | --- | --- | --- | --- | --- | --- | --- | --- | --- |
| 0.53 (-0.93, 2.02) | OT + A-tDCS |  |  |  |  |  |  |  |  |  |  |  |  |
| **1.19 (0.1, 2.26)** | 0.66 (-0.85, 2.14) | OT + Chelating agent |  |  |  |  |  |  |  |  |  |  |  |
| **1.36 (0.29, 2.44)** | 0.82 (-0.56, 2.21) | 0.17 (-0.9, 1.29) | OT + PEA-LUT |  |  |  |  |  |  |  |  |  |  |
| 1.47 (-0.07, 3.04) | 0.94 (-0.85, 2.74) | 0.28 (-1.27, 1.89) | 0.11 (-1.01, 1.24) | PEA-LUT |  |  |  |  |  |  |  |  |  |
| **1.65 (0.51, 2.8)** | 1.12 (-0.32, 2.54) | 0.46 (-0.68, 1.65) | 0.29 (-0.49, 1.07) | 0.18 (-1.2, 1.55) | OT + PEA-LUT + ALA |  |  |  |  |  |  |  |  |
| **2.1 (0.91, 3.29)** | 1.56 (-0.11, 3.22) | 0.9 (-0.41, 2.27) | 0.74 (-0.61, 2.07) | 0.63 (-1.13, 2.36) | 0.45 (-0.95, 1.83) | Forskolin |  |  |  |  |  |  |  |
| **2.3 (1.32, 3.29)** | **1.77 (0.23, 3.29)** | 1.11 (-0.04, 2.29) | 0.94 (-0.22, 2.1) | 0.83 (-0.79, 2.45) | 0.65 (-0.57, 1.87) | 0.21 (-1.07, 1.46) | PRP |  |  |  |  |  |  |
| **2.35 (1.42, 3.29)** | **1.81 (0.5, 3.12)** | **1.16 (0.18, 2.17)** | **0.99 (0.14, 1.83)** | 0.88 (-0.55, 2.28) | 0.7 (-0.24, 1.62) | 0.25 (-0.97, 1.49) | 0.04 (-0.99, 1.09) | OT + Corticosteroid |  |  |  |  |  |
| **2.48 (1.1, 3.9)** | **1.95 (0.32, 3.59)** | 1.3 (-0.09, 2.72) | 1.13 (-0.16, 2.42) | 1.01 (-0.7, 2.73) | 0.83 (-0.51, 2.19) | 0.39 (-1.22, 2) | 0.18 (-1.27, 1.64) | 0.14 (-1.08, 1.37) | OT + Omega-3 |  |  |  |  |
| **2.52 (1.38, 3.67)** | **1.98 (0.55, 3.4)** | **1.32 (0.17, 2.52)** | **1.16 (0.37, 1.94)** | 1.04 (-0.34, 2.41) | **0.87 (0.06, 1.68)** | 0.42 (-0.97, 1.82) | 0.21 (-1.01, 1.45) | 0.16 (-0.75, 1.1) | 0.03 (-1.31, 1.38) | OT+ALA |  |  |  |
| **2.65 (1.27, 4.07)** | **2.12 (0.29, 3.94)** | 1.46 (-0.05, 3.02) | 1.3 (-0.23, 2.83) | 1.18 (-0.72, 3.08) | 1.01 (-0.57, 2.58) | 0.56 (-1.06, 2.16) | 0.35 (-1.1, 1.82) | 0.31 (-1.12, 1.74) | 0.17 (-1.6, 1.93) | 0.14 (-1.44, 1.72) | FSPR |  |  |
| **2.57 (1.73, 3.42)** | **2.03 (0.84, 3.24)** | **1.37 (0.52, 2.27)** | **1.21 (0.54, 1.87)** | 1.09 (-0.22, 2.41) | **0.92 (0.14, 1.69)** | 0.47 (-0.69, 1.64) | 0.26 (-0.68, 1.22) | 0.22 (-0.29, 0.74) | 0.08 (-1.03, 1.19) | 0.05 (-0.73, 0.83) | -0.09 (-1.47, 1.29) | OT |  |
| **2.99 (2.38, 3.62)** | **2.46 (1.12, 3.79)** | **1.8 (0.93, 2.7)** | **1.63 (0.76, 2.51)** | **1.52 (0.09, 2.95)** | **1.34 (0.38, 2.29)** | 0.89 (-0.12, 1.91) | 0.69 (-0.07, 1.45) | 0.65 (-0.05, 1.34) | 0.51 (-0.75, 1.75) | 0.48 (-0.49, 1.44) | 0.34 (-0.92, 1.59) | 0.43 (-0.15, 1) | Control |

Note: OT, Olfactory Training; A-tDCS, Anodal Transcranial Direct Current Stimulation; PEA-LUT, Palmitoylethanolamide and Luteolin; PRP, Platelet-Rich Plasma; ALA, Alpha-Lipoic Acid; FSPR, Functional Septorhinoplasty.

**(b) League table for total TDI after exclusion of Abdelazim b 2022 in the leave-one-out sensitivity analysis.**

| **Chelating agent** |  |  |  |  |  |  |  |  |  |  |  |  |  |
| --- | --- | --- | --- | --- | --- | --- | --- | --- | --- | --- | --- | --- | --- |
| 1.37 (-0.95, 3.84) | OT + A-tDCS |  |  |  |  |  |  |  |  |  |  |  |  |
| 1.98 (0.27, 3.83) | 0.61 (-1.85, 3.02) | OT + Chelating agent |  |  |  |  |  |  |  |  |  |  |  |
| 2.19 (0.48, 4.07) | 0.82 (-1.43, 3.08) | 0.2 (-1.59, 2.04) | OT + PEA-LUT |  |  |  |  |  |  |  |  |  |  |
| 2.3 (-0.23, 4.99) | 0.93 (-2.02, 3.9) | 0.32 (-2.33, 2.98) | 0.11 (-1.79, 2.03) | PEA-LUT |  |  |  |  |  |  |  |  |  |
| 2.48 (0.67, 4.49) | 1.12 (-1.21, 3.46) | 0.5 (-1.41, 2.46) | 0.29 (-1, 1.6) | 0.18 (-2.13, 2.51) | OT + PEA-LUT + ALA |  |  |  |  |  |  |  |  |
| 2.93 (0.89, 5.14) | 1.57 (-1.28, 4.41) | 0.95 (-1.38, 3.32) | 0.74 (-1.63, 3.06) | 0.63 (-2.4, 3.66) | 0.45 (-2, 2.89) | Forskolin |  |  |  |  |  |  |  |
| 3.12 (1.51, 4.89) | 1.75 (-0.8, 4.3) | 1.14 (-0.82, 3.11) | 0.93 (-1.05, 2.9) | 0.82 (-1.96, 3.57) | 0.64 (-1.46, 2.72) | 0.19 (-2.08, 2.45) | PRP |  |  |  |  |  |  |
| 3.18 (1.7, 4.8) | 1.81 (-0.33, 3.96) | 1.19 (-0.43, 2.84) | 0.99 (-0.44, 2.4) | 0.88 (-1.5, 3.25) | 0.7 (-0.89, 2.24) | 0.24 (-1.94, 2.43) | 0.05 (-1.7, 1.81) | OT + Corticosteroid |  |  |  |  |  |
| 3.32 (1.05, 5.76) | 1.96 (-0.78, 4.68) | 1.34 (-1.05, 3.75) | 1.14 (-1.1, 3.34) | 1.02 (-1.88, 3.95) | 0.84 (-1.47, 3.13) | 0.39 (-2.44, 3.21) | 0.2 (-2.3, 2.74) | 0.15 (-1.97, 2.24) | OT+Omega-3 |  |  |  |  |
| 3.35 (1.53, 5.35) | 1.99 (-0.34, 4.34) | 1.37 (-0.55, 3.32) | 1.17 (-0.14, 2.48) | 1.05 (-1.26, 3.38) | 0.87 (-0.49, 2.22) | 0.42 (-2.01, 2.88) | 0.23 (-1.83, 2.34) | 0.17 (-1.38, 1.77) | 0.03 (-2.27, 2.35) | OT+ALA |  |  |  |
| 3.49 (1.33, 5.81) | 2.12 (-0.85, 5.07) | 1.51 (-0.92, 3.97) | 1.3 (-1.16, 3.75) | 1.19 (-1.93, 4.3) | 1 (-1.55, 3.54) | 0.56 (-2.17, 3.27) | 0.37 (-2.03, 2.76) | 0.31 (-1.97, 2.59) | 0.16 (-2.74, 3.07) | 0.13 (-2.43, 2.68) | FSPR |  |  |
| 3.41 (2.08, 4.9) | 2.04 (0.09, 3.99) | 1.42 (0.01, 2.89) | 1.22 (0.1, 2.33) | 1.11 (-1.1, 3.32) | 0.92 (-0.38, 2.21) | 0.47 (-1.59, 2.56) | 0.28 (-1.34, 1.92) | 0.23 (-0.65, 1.11) | 0.08 (-1.82, 2) | 0.05 (-1.25, 1.35) | -0.08 (-2.26, 2.11) | OT |  |
| 3.83 (2.86, 4.93) | 2.46 (0.3, 4.62) | 1.84 (0.41, 3.3) | 1.63 (0.17, 3.08) | 1.53 (-0.88, 3.92) | 1.34 (-0.26, 2.91) | 0.89 (-0.95, 2.74) | 0.71 (-0.63, 2.04) | 0.65 (-0.5, 1.79) | 0.5 (-1.62, 2.63) | 0.47 (-1.14, 2.07) | 0.33 (-1.64, 2.31) | 0.42 (-0.53, 1.36) | Control |

Note: OT, Olfactory Training; A-tDCS, Anodal Transcranial Direct Current Stimulation; PEA-LUT, Palmitoylethanolamide and Luteolin; PRP, Platelet-Rich Plasma; ALA, Alpha-Lipoic Acid; FSPR, Functional Septorhinoplasty.

**(c) League table for total TDI after exclusion of Abdelazim c 2022 in the leave-one-out sensitivity analysis.**

| **Chelating agent** |  |  |  |  |  |  |  |  |  |  |  |  |  |
| --- | --- | --- | --- | --- | --- | --- | --- | --- | --- | --- | --- | --- | --- |
| 1.35 (-1, 3.86) | OT + A-tDCS |  |  |  |  |  |  |  |  |  |  |  |  |
| 1.95 (0.19, 3.83) | 0.61 (-1.89, 3.05) | OT + Chelating agent |  |  |  |  |  |  |  |  |  |  |  |
| 2.15 (0.43, 4.05) | 0.81 (-1.47, 3.1) | 0.21 (-1.63, 2.1) | OT + PEA-LUT |  |  |  |  |  |  |  |  |  |  |
| 2.26 (-0.31, 5.03) | 0.93 (-2.08, 3.91) | 0.32 (-2.38, 3.03) | 0.11 (-1.84, 2.06) | PEA-LUT |  |  |  |  |  |  |  |  |  |
| 2.46 (0.61, 4.5) | 1.12 (-1.27, 3.5) | 0.51 (-1.44, 2.51) | 0.3 (-1.01, 1.62) | 0.19 (-2.17, 2.55) | OT + PEA-LUT + ALA |  |  |  |  |  |  |  |  |
| 2.89 (0.84, 5.14) | 1.55 (-1.33, 4.42) | 0.95 (-1.42, 3.34) | 0.74 (-1.67, 3.12) | 0.62 (-2.46, 3.71) | 0.44 (-2.06, 2.91) | Forskolin |  |  |  |  |  |  |  |
| 3.08 (1.48, 4.87) | 1.74 (-0.85, 4.32) | 1.14 (-0.86, 3.16) | 0.93 (-1.07, 2.92) | 0.82 (-1.99, 3.62) | 0.63 (-1.5, 2.74) | 0.19 (-2.12, 2.51) | PRP |  |  |  |  |  |  |
| 3.15 (1.65, 4.81) | 1.8 (-0.39, 3.97) | 1.2 (-0.46, 2.88) | 0.99 (-0.47, 2.43) | 0.88 (-1.58, 3.29) | 0.69 (-0.91, 2.28) | 0.25 (-1.96, 2.47) | 0.06 (-1.73, 1.86) | OT + Corticosteroid |  |  |  |  |  |
| 3.29 (0.98, 5.78) | 1.96 (-0.83, 4.7) | 1.35 (-1.08, 3.78) | 1.14 (-1.12, 3.36) | 1.03 (-1.98, 3.99) | 0.84 (-1.53, 3.15) | 0.39 (-2.46, 3.28) | 0.2 (-2.34, 2.77) | 0.15 (-2.01, 2.29) | OT + Omega-3 |  |  |  |  |
| 3.33 (1.48, 5.36) | 1.99 (-0.41, 4.36) | 1.38 (-0.58, 3.38) | 1.17 (-0.15, 2.5) | 1.07 (-1.3, 3.42) | 0.87 (-0.51, 2.26) | 0.43 (-2.04, 2.93) | 0.24 (-1.86, 2.37) | 0.19 (-1.42, 1.8) | 0.04 (-2.31, 2.4) | OT+ALA |  |  |  |
| 3.45 (1.25, 5.81) | 2.1 (-0.88, 5.09) | 1.5 (-0.97, 4.03) | 1.29 (-1.2, 3.82) | 1.17 (-1.98, 4.36) | 0.99 (-1.6, 3.59) | 0.55 (-2.17, 3.3) | 0.36 (-2.02, 2.79) | 0.31 (-2.03, 2.65) | 0.16 (-2.78, 3.1) | 0.12 (-2.49, 2.72) | FSPR |  |  |
| 3.38 (2.04, 4.89) | **2.03 (0.05, 4.01)** | 1.43 (-0.02, 2.93) | **1.22 (0.09, 2.36)** | 1.11 (-1.15, 3.35) | 0.92 (-0.4, 2.24) | 0.48 (-1.61, 2.6) | 0.29 (-1.36, 1.96) | 0.23 (-0.66, 1.14) | 0.08 (-1.84, 2.04) | 0.05 (-1.28, 1.37) | -0.07 (-2.3, 2.16) | OT |  |
| 3.79 (2.82, 4.92) | **2.44 (0.24, 4.63)** | **1.84 (0.37, 3.33)** | **1.63 (0.15, 3.11)** | 1.52 (-0.93, 3.96) | 1.32 (-0.31, 2.96) | 0.89 (-0.98, 2.78) | 0.7 (-0.65, 2.05) | 0.64 (-0.54, 1.82) | 0.49 (-1.67, 2.65) | 0.46 (-1.19, 2.08) | 0.34 (-1.69, 2.34) | 0.41 (-0.55, 1.36) | Control |

Note: OT, Olfactory Training; A-tDCS, Anodal Transcranial Direct Current Stimulation; PEA-LUT, Palmitoylethanolamide and Luteolin; PRP, Platelet-Rich Plasma; ALA, Alpha-Lipoic Acid; FSPR, Functional Septorhinoplasty.

**(d) League table for total TDI after exclusion of Abdelazim 2023 in the leave-one-out sensitivity analysis.**

| **Chelating agent** |  |  |  |  |  |  |  |  |  |  |  |  |  |
| --- | --- | --- | --- | --- | --- | --- | --- | --- | --- | --- | --- | --- | --- |
| 1.33 (-0.84, 3.69) | OT + A-tDCS |  |  |  |  |  |  |  |  |  |  |  |  |
| 0.98 (-1.02, 3.13) | -0.36 (-3.18, 2.43) | OT + Chelating agent |  |  |  |  |  |  |  |  |  |  |  |
| **2.14 (0.56, 3.91)** | 0.81 (-1.32, 2.95) | 1.17 (-1.19, 3.54) | OT + PEA-LUT |  |  |  |  |  |  |  |  |  |  |
| 2.25 (-0.13, 4.8) | 0.93 (-1.87, 3.71) | 1.27 (-1.69, 4.28) | 0.11 (-1.7, 1.93) | PEA-LUT |  |  |  |  |  |  |  |  |  |
| **2.44 (0.76, 4.33)** | 1.11 (-1.12, 3.34) | 1.47 (-0.98, 3.94) | 0.3 (-0.93, 1.54) | 0.19 (-1.99, 2.38) | OT + PEA-LUT + ALA |  |  |  |  |  |  |  |  |
| **2.69 (0.84, 4.73)** | 1.36 (-1.38, 4.05) | 1.72 (-0.84, 4.28) | 0.55 (-1.73, 2.77) | 0.44 (-2.48, 3.32) | 0.26 (-2.1, 2.56) | Forskolin |  |  |  |  |  |  |  |
| **2.88 (1.44, 4.52)** | 1.56 (-0.88, 3.97) | 1.91 (-0.36, 4.17) | 0.74 (-1.17, 2.64) | 0.63 (-2, 3.24) | 0.44 (-1.55, 2.45) | 0.19 (-1.97, 2.35) | PRP |  |  |  |  |  |  |
| **3.1 (1.75, 4.6)** | 1.76 (-0.29, 3.78) | 2.12 (-0.07, 4.31) | 0.96 (-0.41, 2.29) | 0.85 (-1.41, 3.07) | 0.66 (-0.85, 2.13) | 0.4 (-1.66, 2.48) | 0.21 (-1.48, 1.88) | OT + Corticosteroid |  |  |  |  |  |
| **3.28 (1.16, 5.6)** | 1.95 (-0.65, 4.53) | 2.31 (-0.45, 5.08) | 1.14 (-0.96, 3.23) | 1.03 (-1.74, 3.78) | 0.84 (-1.35, 3) | 0.59 (-2.08, 3.26) | 0.4 (-1.99, 2.77) | 0.19 (-1.8, 2.18) | OT + Omega-3 |  |  |  |  |
| **3.31 (1.61, 5.21)** | 1.98 (-0.25, 4.22) | 2.34 (-0.09, 4.82) | 1.17 (-0.06, 2.41) | 1.06 (-1.12, 3.24) | 0.87 (-0.41, 2.16) | 0.62 (-1.72, 2.97) | 0.43 (-1.56, 2.44) | 0.21 (-1.26, 1.73) | 0.03 (-2.15, 2.23) | OT+ALA |  |  |  |
| **3.25 (1.25, 5.43)** | 1.92 (-0.92, 4.74) | 2.27 (-0.39, 4.94) | 1.11 (-1.27, 3.45) | 1 (-1.99, 3.96) | 0.81 (-1.63, 3.24) | 0.56 (-2, 3.11) | 0.37 (-1.91, 2.63) | 0.16 (-2.03, 2.36) | -0.04 (-2.8, 2.75) | -0.06 (-2.52, 2.36) | FSPR |  |  |
| **3.36 (2.16, 4.74)** | **2.03 (0.16, 3.87)** | **2.38 (0.27, 4.5)** | **1.22 (0.15, 2.27)** | 1.11 (-0.99, 3.19) | 0.92 (-0.31, 2.14) | 0.66 (-1.31, 2.66) | 0.47 (-1.12, 2.04) | 0.26 (-0.56, 1.1) | 0.08 (-1.73, 1.87) | 0.05 (-1.2, 1.27) | 0.11 (-1.99, 2.24) | OT |  |
| **3.58 (2.78, 4.54)** | **2.25 (0.15, 4.32)** | **2.61 (0.73, 4.49)** | **1.44 (0, 2.85)** | 1.33 (-0.98, 3.62) | 1.14 (-0.43, 2.68) | 0.89 (-0.85, 2.64) | 0.7 (-0.57, 1.95) | 0.48 (-0.63, 1.6) | 0.3 (-1.73, 2.33) | 0.27 (-1.31, 1.81) | 0.33 (-1.55, 2.22) | 0.22 (-0.73, 1.17) | Control |

Note: OT, Olfactory Training; A-tDCS, Anodal Transcranial Direct Current Stimulation; PEA-LUT, Palmitoylethanolamide and Luteolin; PRP, Platelet-Rich plasma; ALA, Alpha-Lipoic Acid; FSPR, Functional Septorhinoplasty;

**(e) League table for total TDI after exclusion of Imam 2023 in the leave-one-out sensitivity analysis.**

| **Chelating agent** |  |  |  |  |  |  |  |  |  |  |  |  |  |
| --- | --- | --- | --- | --- | --- | --- | --- | --- | --- | --- | --- | --- | --- |
| 1.16 (-1.08, 3.49) | OT + A-tDCS |  |  |  |  |  |  |  |  |  |  |  |  |
| 1.76 (0.16, 3.47) | 0.61 (-1.72, 2.96) | OT + Chelating agent |  |  |  |  |  |  |  |  |  |  |  |
| 1.97 (0.36, 3.71) | 0.82 (-1.37, 3) | 0.2 (-1.57, 1.99) | OT + PEA-LUT |  |  |  |  |  |  |  |  |  |  |
| 2.08 (-0.36, 4.66) | 0.93 (-1.93, 3.8) | 0.32 (-2.24, 2.86) | 0.11 (-1.75, 1.96) | PEA-LUT |  |  |  |  |  |  |  |  |  |
| 2.27 (0.54, 4.12) | 1.12 (-1.16, 3.4) | 0.5 (-1.39, 2.39) | 0.3 (-0.97, 1.56) | 0.19 (-2.06, 2.44) | OT + PEA-LUT + ALA |  |  |  |  |  |  |  |  |
| 2.7 (0.79, 4.78) | 1.55 (-1.17, 4.31) | 0.94 (-1.32, 3.24) | 0.74 (-1.53, 3.04) | 0.62 (-2.29, 3.56) | 0.44 (-1.92, 2.82) | Forskolin |  |  |  |  |  |  |  |
| 2.9 (1.4, 4.52) | 1.75 (-0.7, 4.23) | 1.14 (-0.77, 3.05) | 0.93 (-1, 2.83) | 0.82 (-1.85, 3.49) | 0.63 (-1.39, 2.64) | 0.19 (-2.02, 2.39) | PRP |  |  |  |  |  |  |
| 2.96 (1.58, 4.46) | 1.8 (-0.27, 3.88) | 1.19 (-0.38, 2.79) | 0.99 (-0.39, 2.38) | 0.88 (-1.43, 3.21) | 0.68 (-0.82, 2.22) | 0.25 (-1.86, 2.35) | 0.05 (-1.63, 1.77) | OT + Corticosteroid |  |  |  |  |  |
| 3.1 (0.96, 5.41) | 1.95 (-0.64, 4.58) | 1.34 (-0.96, 3.67) | 1.13 (-0.97, 3.28) | 1.02 (-1.77, 3.86) | 0.83 (-1.36, 3.06) | 0.4 (-2.29, 3.12) | 0.2 (-2.2, 2.63) | 0.15 (-1.87, 2.18) | OT + Omega-3 |  |  |  |  |
| 3.14 (1.4, 5.01) | 1.98 (-0.28, 4.26) | 1.37 (-0.51, 3.27) | 1.17 (-0.09, 2.44) | 1.06 (-1.18, 3.3) | 0.87 (-0.45, 2.2) | 0.43 (-1.93, 2.79) | 0.24 (-1.77, 2.26) | 0.18 (-1.34, 1.73) | 0.03 (-2.2, 2.24) | OT+ALA |  |  |  |
| 3.26 (1.2, 5.43) | 2.11 (-0.73, 4.96) | 1.49 (-0.9, 3.88) | 1.28 (-1.12, 3.66) | 1.17 (-1.85, 4.2) | 0.99 (-1.5, 3.46) | 0.55 (-2.08, 3.16) | 0.35 (-1.96, 2.68) | 0.3 (-1.94, 2.52) | 0.16 (-2.69, 2.95) | 0.12 (-2.37, 2.59) | FSPR |  |  |
| 3.19 (1.97, 4.54) | **2.03 (0.14, 3.94)** | **1.42 (0.03, 2.83)** | **1.21 (0.13, 2.31)** | 1.11 (-1.04, 3.26) | 0.92 (-0.33, 2.18) | 0.48 (-1.53, 2.49) | 0.28 (-1.28, 1.88) | 0.23 (-0.63, 1.09) | 0.08 (-1.75, 1.91) | 0.05 (-1.23, 1.3) | -0.08 (-2.19, 2.08) | OT |  |
| 3.61 (2.78, 4.56) | **2.45 (0.36, 4.57)** | **1.84 (0.45, 3.25)** | **1.63 (0.21, 3.05)** | 1.52 (-0.82, 3.86) | 1.33 (-0.22, 2.89) | 0.89 (-0.9, 2.69) | 0.7 (-0.58, 2) | 0.64 (-0.47, 1.77) | 0.5 (-1.55, 2.52) | 0.46 (-1.11, 2.02) | 0.34 (-1.57, 2.29) | 0.42 (-0.5, 1.33) | Control |

Note: OT, Olfactory Training; A-tDCS, Anodal Transcranial Direct Current Stimulation; PEA-LUT, Palmitoylethanolamide and Luteolin; PRP, Platelet-Rich Plasma; ALA, Alpha-Lipoic Acid; FSPR, Functional Septorhinoplasty;

**(f) League table for total TDI after exclusion of Altemani 2024 in the leave-one-out sensitivity analysis.**

| **Chelating agent** |  |  |  |  |  |  |  |  |  |  |  |  |  |
| --- | --- | --- | --- | --- | --- | --- | --- | --- | --- | --- | --- | --- | --- |
| 1.15 (-1.07, 3.53) | OT + A-tDCS |  |  |  |  |  |  |  |  |  |  |  |  |
| 1.76 (0.14, 3.48) | 0.61 (-1.77, 2.95) | OT + Chelating agent |  |  |  |  |  |  |  |  |  |  |  |
| 1.96 (0.36, 3.69) | 0.82 (-1.35, 2.96) | 0.2 (-1.55, 1.99) | OT + PEA-LUT |  |  |  |  |  |  |  |  |  |  |
| 2.07 (-0.36, 4.63) | 0.91 (-1.93, 3.79) | 0.3 (-2.23, 2.9) | 0.1 (-1.73, 1.97) | PEA-LUT |  |  |  |  |  |  |  |  |  |
| 2.26 (0.54, 4.13) | 1.12 (-1.19, 3.38) | 0.5 (-1.36, 2.4) | 0.3 (-0.97, 1.55) | 0.2 (-2.07, 2.44) | OT + PEA-LUT + ALA |  |  |  |  |  |  |  |  |
| 2.71 (0.76, 4.76) | 1.55 (-1.2, 4.29) | 0.95 (-1.3, 3.21) | 0.75 (-1.55, 3) | 0.64 (-2.3, 3.57) | 0.45 (-1.92, 2.79) | Forskolin |  |  |  |  |  |  |  |
| 2.9 (1.4, 4.51) | 1.75 (-0.71, 4.21) | 1.14 (-0.76, 3.06) | 0.93 (-0.98, 2.83) | 0.83 (-1.85, 3.48) | 0.64 (-1.38, 2.64) | 0.19 (-1.99, 2.39) | PRP |  |  |  |  |  |  |
| 2.95 (1.59, 4.43) | 1.8 (-0.28, 3.87) | 1.19 (-0.39, 2.8) | 0.99 (-0.39, 2.36) | 0.89 (-1.43, 3.19) | 0.69 (-0.84, 2.21) | 0.24 (-1.85, 2.36) | 0.05 (-1.64, 1.76) | OT + Corticosteroid |  |  |  |  |  |
| 3.1 (0.94, 5.4) | 1.95 (-0.7, 4.57) | 1.35 (-0.95, 3.67) | 1.14 (-0.98, 3.26) | 1.04 (-1.79, 3.88) | 0.85 (-1.38, 3.07) | 0.39 (-2.3, 3.12) | 0.21 (-2.2, 2.63) | 0.15 (-1.86, 2.18) | OT + Omega-3 |  |  |  |  |
| 3.14 (1.42, 4.99) | 1.99 (-0.3, 4.24) | 1.38 (-0.5, 3.27) | 1.17 (-0.1, 2.44) | 1.07 (-1.18, 3.3) | 0.87 (-0.44, 2.19) | 0.43 (-1.93, 2.79) | 0.24 (-1.76, 2.26) | 0.18 (-1.32, 1.71) | 0.03 (-2.18, 2.24) | OT+ALA |  |  |  |
| 3.25 (1.18, 5.45) | 2.11 (-0.76, 4.94) | 1.5 (-0.89, 3.87) | 1.29 (-1.12, 3.69) | 1.19 (-1.85, 4.21) | 0.99 (-1.49, 3.47) | 0.55 (-2.08, 3.19) | 0.36 (-1.96, 2.66) | 0.3 (-1.92, 2.54) | 0.15 (-2.68, 2.96) | 0.12 (-2.36, 2.6) | FSPR |  |  |
| 3.18 (1.97, 4.52) | **2.03 (0.13, 3.91)** | **1.42 (0.04, 2.84)** | **1.22 (0.14, 2.3)** | 1.12 (-1.03, 3.26) | 0.92 (-0.33, 2.18) | 0.48 (-1.52, 2.49) | 0.28 (-1.28, 1.87) | 0.23 (-0.62, 1.09) | 0.08 (-1.75, 1.9) | 0.05 (-1.21, 1.31) | -0.07 (-2.2, 2.06) | OT |  |
| 3.6 (2.77, 4.55) | **2.45 (0.33, 4.53)** | **1.83 (0.45, 3.26)** | **1.63 (0.23, 3.03)** | 1.53 (-0.81, 3.86) | 1.34 (-0.21, 2.89) | 0.89 (-0.87, 2.68) | 0.7 (-0.58, 1.99) | 0.65 (-0.46, 1.77) | 0.5 (-1.53, 2.54) | 0.46 (-1.08, 2.01) | 0.34 (-1.59, 2.28) | 0.42 (-0.49, 1.33) | Control |

Note: OT, Olfactory Training; A-tDCS, Anodal Transcranial Direct Current Stimulation; PEA-LUT, Palmitoylethanolamide and Luteolin; PRP, Platelet-Rich Plasma; ALA, Alpha-Lipoic Acid; FSPR, Functional Septorhinoplasty.

**(g) League table for total TDI after exclusion of Imam 2025 in the leave-one-out sensitivity analysis.**

| **Chelating agent** |  |  |  |  |  |  |  |  |  |  |  |  |  |
| --- | --- | --- | --- | --- | --- | --- | --- | --- | --- | --- | --- | --- | --- |
| 1.15 (-1.05, 3.52) | OT + A-tDCS |  |  |  |  |  |  |  |  |  |  |  |  |
| 1.76 (0.14, 3.5) | 0.61 (-1.78, 2.96) | OT + Chelating agent |  |  |  |  |  |  |  |  |  |  |  |
| 1.97 (0.36, 3.71) | 0.82 (-1.37, 3.02) | 0.21 (-1.54, 2) | OT + PEA-LUT |  |  |  |  |  |  |  |  |  |  |
| 2.07 (-0.36, 4.63) | 0.92 (-1.95, 3.79) | 0.31 (-2.25, 2.91) | 0.11 (-1.76, 1.96) | PEA-LUT |  |  |  |  |  |  |  |  |  |
| 2.27 (0.53, 4.14) | 1.12 (-1.17, 3.4) | 0.5 (-1.36, 2.42) | 0.29 (-0.97, 1.56) | 0.19 (-2.06, 2.44) | OT + PEA-LUT + ALA |  |  |  |  |  |  |  |  |
| 2.7 (0.77, 4.76) | 1.55 (-1.22, 4.31) | 0.94 (-1.33, 3.22) | 0.73 (-1.57, 2.99) | 0.63 (-2.31, 3.56) | 0.44 (-1.94, 2.79) | Forskolin |  |  |  |  |  |  |  |
| 2.9 (1.39, 4.54) | 1.76 (-0.74, 4.23) | 1.14 (-0.77, 3.06) | 0.93 (-1.02, 2.84) | 0.83 (-1.86, 3.49) | 0.63 (-1.41, 2.67) | 0.2 (-2, 2.42) | PRP |  |  |  |  |  |  |
| 2.95 (1.57, 4.45) | 1.8 (-0.31, 3.89) | 1.19 (-0.39, 2.8) | 0.98 (-0.4, 2.36) | 0.88 (-1.43, 3.19) | 0.68 (-0.85, 2.21) | 0.25 (-1.86, 2.36) | 0.05 (-1.65, 1.77) | OT + Corticosteroid |  |  |  |  |  |
| 3.1 (0.92, 5.42) | 1.95 (-0.71, 4.6) | 1.34 (-0.96, 3.68) | 1.13 (-1, 3.26) | 1.03 (-1.79, 3.83) | 0.84 (-1.4, 3.05) | 0.4 (-2.33, 3.13) | 0.2 (-2.23, 2.63) | 0.15 (-1.87, 2.18) | OT + Omega-3 |  |  |  |  |
| 3.14 (1.39, 5.02) | 1.98 (-0.29, 4.29) | 1.38 (-0.51, 3.28) | 1.17 (-0.1, 2.45) | 1.06 (-1.17, 3.34) | 0.87 (-0.45, 2.18) | 0.44 (-1.94, 2.83) | 0.24 (-1.79, 2.28) | 0.19 (-1.35, 1.73) | 0.04 (-2.19, 2.28) | OT+ALA |  |  |  |
| 3.26 (1.19, 5.49) | 2.11 (-0.75, 4.97) | 1.5 (-0.88, 3.91) | 1.29 (-1.1, 3.69) | 1.18 (-1.86, 4.2) | 1 (-1.49, 3.47) | 0.55 (-2.1, 3.22) | 0.35 (-1.97, 2.7) | 0.31 (-1.91, 2.53) | 0.15 (-2.65, 2.97) | 0.12 (-2.36, 2.59) | FSPR |  |  |
| 3.18 (1.97, 4.54) | **2.03 (0.11, 3.94)** | **1.42 (0.04, 2.84)** | **1.21 (0.12, 2.29)** | 1.11 (-1.03, 3.25) | 0.92 (-0.36, 2.17) | 0.48 (-1.53, 2.49) | 0.28 (-1.29, 1.87) | 0.23 (-0.62, 1.09) | 0.08 (-1.76, 1.92) | 0.05 (-1.23, 1.31) | -0.08 (-2.21, 2.06) | OT |  |
| 3.6 (2.77, 4.57) | **2.45 (0.32, 4.56)** | **1.84 (0.42, 3.26)** | **1.63 (0.21, 3.04)** | 1.52 (-0.81, 3.85) | 1.33 (-0.24, 2.88) | 0.9 (-0.89, 2.69) | 0.7 (-0.6, 2) | 0.64 (-0.46, 1.77) | 0.49 (-1.58, 2.54) | 0.46 (-1.11, 2.01) | 0.34 (-1.61, 2.27) | 0.42 (-0.5, 1.33) | Control |

Note: OT, Olfactory Training; A-tDCS, Anodal transcranial Direct Current Stimulation; PEA-LUT, Palmitoylethanolamide and Luteolin; PRP, Platelet-Rich Plasma; ALA, Alpha-Lipoic Acid; FSPR, Functional Septorhinoplasty.

**Figure S1** Deviance Information Criterion (DIC).

**Figure S2** Inconsistency analyses.

**Figure S3** Heterogeneity test.

**Figure S4** Results of the network meta-analysis. (a) The pairwise comparison network of the three sub-dimensions of TDI; (b) SUCRA probability ranking of the three sub-dimensions of TDI (line chart).

**Figure S5** SUCRA plot.

**Figure S6** Funnel plot.
